# Community mobilization to strengthen support for appropriate and timely use of antenatal and postnatal care: A review of reviews

**DOI:** 10.7189/jogh.11.04076

**Published:** 2021-12-30

**Authors:** Sara Dada, Özge Tunçalp, Anayda Portela, María Barreix, Brynne Gilmore

**Affiliations:** 1 UCD Centre for Interdisciplinary Research, Education and Innovation in Health Systems, School of Nursing, Midwifery and Health Systems, University College Dublin, Dublin, Ireland; 2 School of Nursing, Midwifery and Health Systems, University College Dublin, Dublin, Ireland; 3 UNDP/UNFPA/UNICEF/WHO/World Bank Special Programme of Research, Development and Research Training in Human Reproduction, Department of Sexual and Reproductive Health and Research, World Health Organization, Geneva, Switzerland; 4 Department of Maternal, Newborn, Child and Adolescent Health and Ageing, World Health Organization, Geneva, Switzerland

## Abstract

**Background:**

Antenatal care (ANC) and postnatal care (PNC) are critical opportunities for women, babies and parents/families to receive quality care and support from health services. Community-based interventions may improve the accessibility, availability, and acceptance of this vital care. For example, community mobilization strategies have been used to involve and collaborate with women, families and communities to improve maternal and newborn health.

**Objective:**

To synthesize existing reviews of evidence on community mobilization strategies that strengthen support for appropriate and timely use of ANC and PNC.

**Methods:**

Six databases (MEDLINE, Embase, CINAHL, PsychINFO, Cochrane Library, PROSPERO) were searched for published reviews that describe community mobilization related strategies for ANC and/or PNC. Reviews were eligible for inclusion if they described any initiatives or strategies targeting the promotion of ANC and/or PNC uptake that included an element of community mobilization in a low- or middle-income country (LMIC), published after 2000. Included reviews were critically appraised according to the Joanna Briggs Institute (JBI) Checklist for Systematic Reviews and Evidence Syntheses. This review of reviews was conducted following JBI guidelines for undertaking and reporting umbrella reviews.

**Results:**

In total 23 papers, representing 22 reviews were included. While all 22 reviews contained some description of community mobilization and ANC/PNC, 13 presented more in-depth details on the community mobilization processes and relevant outcomes. Seventeen reviews focused on ANC, four considered both ANC and PNC, and only one focused on PNC. Overall, 16 reviews reported at least one positive association between community mobilization activities and ANC/PNC uptake, while five reviews presented primary studies with no statistically significant change in ANC uptake and one included a primary study with a decrease in use of antenatal facilities. The community mobilization activities described by the reviews ranged from informative, passive communication to more active, participatory approaches that included engaging individuals or consulting local leaders and community members to develop priorities and action plans.

**Conclusions:**

While there is considerable momentum around incorporating community mobilization activities in maternal and newborn health programs, such as improving community support for the uptake of ANC and PNC, there is limited evidence on the processes used. Furthermore, the spectrum of terminology and variation in definitions should be harmonized to guide the implementation and evaluation efforts.

Antenatal care (ANC) and postnatal care (PNC) are critical opportunities for women, babies, parents/caregivers and families to receive quality care. A global study on ANC found significant interregional and intercountry inequalities in ANC service utilization, especially in Asia and Africa [1]. In 2016, the World Health Organization (WHO) released updated recommendations for routine ANC within the continuum of care to improve maternal and newborn health [2]. The WHO recommendations for PNC, published in 2013, are currently being updated [3,4]. With global priorities to improve the availability, accessibility and acceptability of this care, a range of studies have investigated the factors that affect the implementation of these guidelines and the utilization of both ANC and PNC services [4-6].

ANC and PNC share several common factors that may affect their uptake and utilization, including providers’ skills, the relationship between health workers and women, local infrastructure such as distance and transportation to facilities, as well as the influence of women and families’ knowledge, attitudes, beliefs and culture norms regarding care [7-12]. As such, the perceptions, support, and involvement of the partner, family and community members are influential [9]. Community mobilization techniques have been used to both support health promotion and educational programming^2^ as well as increase acceptability and accessibility to the interventions [13].

While the definition of community mobilization varies in the literature, it can largely be described as involving community members in activities to increase support and actions for an intended cause [14,15]. There is a spectrum of activities and approaches to mobilize communities [16]. WHO guidelines for maternal and newborn health (MNH) also describe community-based interventions to improve communication and support and different community mobilization strategies including participatory learning and action (PLA) cycles [6,17,18]. Multiples studies and reviews have been conducted on community mobilization or participation interventions to promote maternal and newborn health and inform international guidelines [6,15,17-19]. These largely center around the effectiveness of such approaches and their influence on outcomes of interest, such as increasing ANC and/or PNC uptake and some report on maternal and newborn morbidity and mortality. Previous systematic reviews on community mobilization for maternal and newborn health have described initiatives such as PLA and women’s groups, as an avenue to provide emotional, social, and psychological support and a cost-effective strategy to improve maternal and neonatal mortality in low-resource settings [20-23]. A larger body of work describes community-based initiatives more broadly, including a range of community mobilization activities [12,22-26].

However, there is also a need to consider the full spectrum of community mobilization activities and implementation processes, and how they improve community support for ANC/PNC. Specifically, the purpose of this review of reviews is therefore to synthesize the existing evidence on what community mobilization strategies are effective in increasing family and community support for appropriate and timely use of ANC and PNC in LMICs. Within these community mobilization strategies, the review also seeks to understand what strategies are used to strengthen family and community support for use of ANC in the first trimester of pregnancy. The findings of this review of reviews are then applied to the 2019 logic model pathway for uptake of ANC services described by Downe et al, which underpins the WHO recommendations on ANC [27].

## METHODS

A systematic review of reviews was conducted in order to gather existing evidence on community mobilization strategies that strengthen support for appropriate and timely use of ANC/PNC, including use of ANC in the first trimester of pregnancy. This approach summarizes the findings of published reviews to synthesize existing evidence in an overview that can inform future guidelines, programmes, and policy [28-30]. As there are already existing systematic reviews on community mobilization within reproductive, maternal, newborn and child health (RMNCH), this methodology was chosen to provide high level evidence on such strategies for ANC/PNC and to reveal where findings are consistent [31,32]. **Table 1** exhibits the operational definitions of the terms used in this review of reviews.

**Table 1:** Operational definitions

Term	Definition
Community Mobilization	Community mobilization interventions “encourage community individuals, groups (including in schools), or organizations to plan, carry out, and evaluate activities on a participatory and sustained basis to improve their health and other needs” [15]. For example, community mobilization includes PLA cycles, community dialogues, and community advocacy activities [15,18].
Antenatal Care (ANC)	“The health care women get while they are pregnant,” [27] includes health screenings, information sharing, counselling, vaccination administration, preventive and curative treatments, and more.
Early ANC	According to WHO guidelines, early ANC is within the first trimester, or often gestational age of ≤12 weeks [6].
Appropriate & timely use of care	Appropriate and timely use of ANC and PNC is based on adhering to country and/or WHO guidelines on the number and timing of contacts.
Postnatal Care (PNC)	The care received by the woman and the newborn after childbirth [3]. The exact definition of the postnatal period can vary, but is often considered the first six weeks after birth [4].

PLA – participatory learning and action, ANC – antenatal care, PNC – postnatal care

### Eligibility criteria

Studies were eligible for inclusion if they described any interventions, initiatives or strategies targeting the promotion of ANC and/or PNC uptake that included an element of community mobilization in a low-and middle-income country (LMIC), as defined by the World Bank Classification at time of study. Eligible reviews of published literature included, but were not limited to, literature reviews, narrative reviews, realist reviews, systematic reviews, scoping reviews, and qualitative evidence syntheses. Only studies published on or after 2000 were included. This year was chosen as a cut-off point in order to capture more relevant research, aligning with the Millennium Development Goals and increased attention on ANC/PNC for maternal, newborn and child health and with the growing focus on community-based interventions such as community mobilization. **Table 2** details the inclusion and exclusion criteria.

**Table 2:** Inclusion/exclusion criteria

Topic	Inclusion criteria	Exclusion criteria
*Population*	Pregnant and postpartum women, companions, birth partners, fathers/caregivers, family members, decision-makers, local authorities/community leaders, community health workers and service providers	
*Exposure/ Intervention*	Initiatives/strategies/interventions targeting promotion of ANC and/or PNC uptake that include an element of community mobilization	No identifiable community mobilization component; use of community health workers or other health providers only to deliver services with no identifiable community mobilization component aimed at increasing ANC/PNC.
*Outcome*	Promotion of ANC/PNC care-seeking behavior; promotion of early ANC	Not focused on promoting ANC/PNC
*Setting*	Focus on LMIC, as defined by World Bank Classification at time of study. Reviews that include studies in high- income countries and LMICs were considered if evidence from LMICs could be extrapolated.	Reviews with no LMICs represented
*Time*	Published on or after 2000	Published before 2000
*Article type*	Reviews of published literature including literature reviews, narrative reviews, realist reviews, systematic reviews, scoping reviews, qualitative syntheses, etc.	Primary, empirical studies; commentaries; abstracts; grey literature
*Language*	All languages included, but search conducted mainly in English.	

ANC – antenatal care, PNC – postnatal care, LMIC – low and middle-income country

### Search strategy

The search strategy was designed around three main concepts: community mobilization, ANC/PNC, and reviews (Appendix S1 in the **Online Supplementary Document**). The search was run across six databases (MEDLINE, Embase, CINAHL, PsychINFO, Cochrane Library, PROSPERO) and snowballing techniques, including examining the bibliographies of included reviews as well as a relevant publication mapping social, behavioral, and community-engagement (SBCE) interventions [15] and key websites (Google Scholar, MASCOT) were conducted. No language restrictions were applied, but the search strategy was run in English.

### Study selection and management

All returns from the database search were imported into Covidence, an online information management system. After managing for duplicates, two reviewers (SD, BG) independently screened the resulting studies’ titles and abstracts. After discussing any conflicts, the reviewers analyzed full texts to determine final inclusion data set, again discussing any conflicts. A third reviewer (AGP) reviewed 20% of the included and excluded articles at full-text stage.

### Data extraction

One reviewer (SD) extracted data from all included studies into an Excel sheet using a pre-developed data extraction tool. Review characteristics and findings relevant to community mobilization and ANC/PNC uptake were considered. Extracted data included: number of studies and geographic range, participants, specific community mobilization strategies used, barriers and facilitators to activity implementation, outcomes reported in the review, recommendations by the review authors, etc. A second reviewer (BG) independently extracted data on approximately 20% of included reviews to compare for inter-rater reliability and resolve any potential discrepancies and also examined all other extracted data for consistency. Data was extracted as it was presented in the published reviews. Reviewers did not extract from the primary sources that were referenced. This led to varying levels of detail in the major findings of reviews as some reported specific outcomes of primary studies while others did not.

### Critical appraisal

Two reviewers (SD, BG) assessed the quality of the included reviews using the Joanna Briggs Institute (JBI) Checklist for Systematic Reviews and Research Syntheses [30,33]. This tool was used due to the range of review methodologies and formats included.

### Data synthesis

Findings from the data extraction were synthesized to answer the research questions previously presented. This was done by collecting the overall conclusions on community mobilization and reported outcomes for ANC and/or PNC utilization, listing the range of mentioned community mobilization strategies and activities used within MNH programmes, and grouping reviews that included community mobilization that impacted on early ANC uptake. In addition to presenting evidence from the included reviews, this review used findings from a recent qualitative evidence synthesis on routine ANC uptake to contextualize some of the practical implications for community mobilization [27]. Downe et al. identified perceptions and experiences of pregnant women and health care providers related to the initial and continued use of ANC and presented these findings following the theory of planned behavior framework [27,34].

## RESULTS

**Figure 1:**
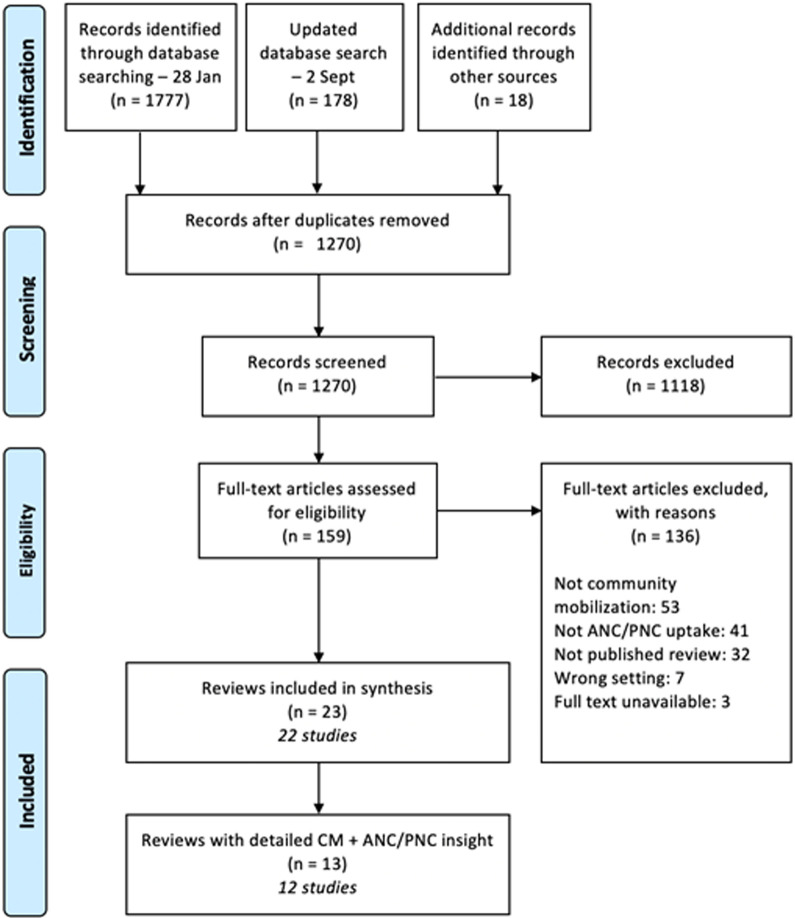
PRISMA screening diagram [35].

Searching occurred between 28 January and 15 February 2021. An updated database search was conducted on 2 September 2021. As highlighted in **Figure 1**, a total of 1955 records were identified during database searching. After removal of duplicates, 1270 articles were screened at title and abstract stage, with 153 full texts screened. An additional 18 resources were reviewed after snowballing, with 6 screened at full-text stage. In total 23 articles [20,21,23,36-55], representing 21 reviews were included, as two of these articles [47,48] were from the same multi-part review and are henceforth referred to as one review.

The JBI Critical Appraisal tool was applied to all included studies and the assessments are available in Appendix S2 in the **Online Supplementary Document****.** No reviews were excluded based on quality, however the challenges reviews faced in terms of quality centered around appropriate and validated appraisal processes as well as clear strategies and sources. Additionally, it is important to note that while all reviews presented included some description of community mobilization and ANC/PNC, 12 articles presented more in-depth details on the community mobilization processes and outcomes relevant to this review of reviews [21,23,39,41-44,47-49,51,53]. This means that the review itself explicitly defined or described how community mobilization was implemented as well as the potential impact those activities had on ANC/PNC uptake. For the remaining 10 reviews, there was limited detail to sufficiently extract data on what community mobilization activity was implemented, though they did report on ANC/PNC uptake.

### Review characteristics

The 22 reviews ranged in size and scope. **Table 3** highlights some key characteristics and details the interventions and findings of interest to this review. This range in reviews was intentional as the search strategy was designed to include reviews of different focuses that contained any element of community mobilization and ANC/PNC uptake. The reviews utilized varying methodologies and included: systematic reviews (n = 11), meta-analyses (n = 3), evidence reviews (n = 3), narrative review (n = 1), integrative review (n = 1), scoping review (n = 1), and general reviews/syntheses (n = 2). Reviews focused on LMIC settings globally (n = 12) [21,23,37-40,43,44,47-50,54], sub-Saharan Africa (n = 4) [20,36,52,53], and South Asia (n = 3) [46,51,55]. Three reviews had no geographic limitations and typically specified findings from high-, middle-, and low-income settings [41,42,45]. In terms of the Sustainable Development Goals (SDG) regions, Central and Southern Asia (n = 18) was the most represented region in the reviews, followed by Sub-Saharan Africa (n = 17), Latin America and the Caribbean (n = 8) and Eastern and South-Eastern Asia (n = 8), Europe and North America (n = 7), Northern Africa and Western Asia (n = 3), and then Oceania (n = 2). Appendix S3 in the **Online Supplementary Document** displays the breakdown in representation across the SDG regions. While the earliest review included was published in 2010, there has been a steady increase in publications on this subject over the last decade. Sixteen of the included reviews were published in or after 2015.

**Table 3:** Summary table*

Review details	Number of studies in review	Geographic setting for review	Review focus & objectives	Community mobilization activities	outcome of interest (number of relevant included studies)	Key findings/ outcomes
George et al[39] 2015 – *Systematic Review*	4	LMICs	Maternal & Newborn Health – *examining how the awareness of interventions that promote awareness of rights affects demand/use of care to affect maternal and newborn health outcomes*	Public meetings, events with community and staff, household visits, women's groups, awareness campaigns, local leader meetings	ANC (4) & early ANC (1)	Four studies demonstrated increase in ANC use with the interventions, though one was not statistically significant.
Kearns et al[41] 2016 - *Review*	8	Global	ANC & PNC – *identifying innovative approaches to improve ANC & PNC*	Women's groups, lady health workers, committees with local leaders	ANC & PNC – 3 total	Women's groups can improve accessibility and acceptability of ANC and PNC by building consensus, creating a support network, and providing relevant health and pregnancy related information.
Kuhlmann et al[42] 2016 – *Review of Evidence*	32	Global	Sexual, Reproductive, & Maternal Health – *assessing the effectiveness of community mobilization interventions*	Home visits for education; peer-facilitated and community/staff led participatory learning groups	ANC (16) & early ANC (1)	Involving community members in interventions led to better outcomes including increases in at least one or more ANC visits and increases in booking early ANC visits.
Lassi et al[23] 2019 – *Systematic Review*	33	LMICs	Newborn Health – *comparing community health educational strategies*	Educational interventions - one-to-one & group counseling	ANC (18)	Analyses in the review demonstrated community health interventions and both one-to-one and group counselling increased ANC utilization.
Mangham-Jeffries et al[43] 2014 – *Systematic Review*	48	LMICs	Maternal & Newborn Health – *determining the cost-effectiveness of strategies to improve maternal and newborn health*	Women's groups, home visits	ANC (2)	Studies reported increased use of ANC as well as new ANC users. Overall, findings suggest that women’s groups are a cost-effective intervention.
Marston et al[44] 2013 – *Systematic Review*	15	LMICs	Maternal & Newborn Health – *examining the effectiveness of community participation interventions in maternal & newborn health*	Women's groups; evidence-based dialogue model; participatory methodologies	ANC (7)	Community participation interventions including women’s groups and participatory dialogues had positive effects on ANC uptake, though these were not always statistically significant. There is a lack of evidence about why these interventions do/do not succeed.
Mbuagbaw et al[45] 2015 – *Systematic Review*	34	Global	ANC & early ANC – *assessing the effects of health system and community interventions to improve ANC*	Mass media campaigns. Social mobilization. Information-education-communication (IEC).	ANC (23) & early ANC (1)	This synthesis considered outcomes when populations received one or multiple community interventions. Community-based interventions to increase antenatal care included media campaigns, education, and financial incentives. There were high levels of heterogeneity, but most of the pooled analyses found increases in ANC coverage/visits in populations that received an intervention or combination of interventions, though this was not always statistically significant.
Perry et al[47,48,56] [3 sources] 2017 – *Review of Evidence*	700	LMICs	Maternal, Newborn, & Child Health – *examining community-based primary health care to improve MNCH*	Home visits, community case management, participatory women's group	ANC (37)	Of the included studies, 34 studies reported increases in ANC, three studies reported no change (or no statistically significant change) in ANC, no studies reported a decrease in ANC [chapter 2].
Prost et al[21] 2013 – *Systematic Review & Meta-Analysis*	7	LMICs	Maternal & Newborn Health – *assessing the effect of women’s groups using PLA*	Women's groups and facilitated PLA cycles	ANC (6) & PNC (5)	Two studies reported on how women's groups significantly increased ANC uptake, whereas four did not find significant differences in ANC uptake. One study reported an increase in postpartum checks. One study also reported a statistically significant increase in care-seeking practices in case of a newborn's health problem. Two studies reported a statistically insignificant increase in care-seeking in case of a postpartum problem for the mother, while four reported a statistically insignificant increase in care-seeking in case of a postpartum problem for the newborn.
Sarkar et al[49] 2015 – *Systematic Review*	8	LMICs	Reproductive Health – *analyzing effective community-based interventions to improve reproductive health*	Individual and group counseling, community education campaigns, group training, advocacy workshops, sensitization, street plays and drama, youth groups, events	ANC & PNC – 5 total	Three of the initiatives demonstrated improvements in ANC including ANC check-ups by first time mothers. These three initiatives also reported improvements in PNC, with two showing significant improvements in first-time mothers receiving PNC check-ups within 6 weeks of postpartum.
Sharma et al[51] 2018 – *Systematic Review*	11	South Asia	Maternal Health – *comparing the effectiveness of interventions to promote family and community participation in maternal health*	Community mobilisers deliver MH education: home care and community care; CHWs community education; women's MH education group; women and men's MH health care education groups	ANC (8)	Community interventions increased attendance of 1+ ANC visit.
Wekesah et al[53] 2016 – *Systematic Review*	73	Sub-Saharan Africa	Maternal Health – *examining the effectiveness of non-drug interventions in improving outcomes in maternal health*	1. Community mobilization through women’s groups;	ANC (6) & PNC (1)	For non-drug interventions such as community mobilization and peer-based programs, one study reported a significant increase in ANC and another study found women in the intervention arm were more likely to attend PNC within the first 48 h.
				2. Skilled birth attendants;		
				3. Training health extension workers;		
				4. Training and deployment of community health development agents;		
				5. Traditional birth attendants (TBAs);		
				6. Family and community members meetings on health care;		
				7. Trained volunteers to provide health care at the community;		
				8. Village health teams;		
				9. Peer mentors who women are living with HIV		
Beck et al [36] 2019 – *Systematic Review*	19	Sub-Saharan Africa	Maternal & Child Health – *understanding how community mobilization interventions for maternal and child health impact empowerment*	None listed	ANC (2)	Two studies demonstrated CM increased care-seeking behaviours, two studies demonstrated increased number of ANC visits.
Deshmukh et al [37] 2020 – *Integrative Review*	47	LMICs	PNC – *identifying the determinants of PNC service provision and utilization*	Social mobilization as an opportunity for education/ awareness	PNC (*unknown)*	The reviewers identified social mobilization as an opportunity to increase awareness of PNC services and health complications in the nexus framework they applied to the included studies.
George & Branchini [38] 2017 – *Synthesis*	26	LMICs	Maternal Health – *examining initiatives that promote awareness of rights to quality maternal care*	Public meetings, committees, information dissemination	ANC (4)	Three studies demonstrated an increase in ANC uptake with community mobilization initiatives present. An additional study demonstrated an increase in ANC uptake but it was not statistically significant.
Gogia et al [40] 2011 – Systematic Review & Meta-Analysis	13	LMICs	Newborn Health – *assessing the effect of community-based neonatal care*	Group meetings, community health committees, education sessions, participatory action learning cycles	ANC (5)	One study demonstrated improvement in at least 1 ANC visit.
Muzyamba et al [20] 2017 – *Systematic Review*	14	Sub-Saharan Africa	Maternal Health for Women with HIV – *reviewing the evidence on the role of community mobilization in maternal care for women with HIV*	Group/peer support for pregnant women/new mothers	ANC (3)	Three studies reported that peer support for women with HIV increased access to health resources, including ANC.
Parsekar et al [46] 2020 – *Narrative Review*	*Not stated*	South Asia	Reproductive, Maternal, Newborn, Adolescent, & Child Health – *collating the evidence on behavior change communication interventions*	Information, education, communication/behavior change communication initiative	ANC (1)	*This narrative review described the strategies used by one IEC initiative in Nepal but did not report on the outcomes.*
Schiffman et al [50] 2010 – *Review of Evidence*	9	LMICs	Perinatal Health *– to describe the effects of community-based intervention packages*	Community health committees, group meetings, participatory learning activities, folksongs	ANC (4)	*This review focused on presenting mortality-related outcomes and did not report on the outcomes related to changes in care seeking.*
Singh et al [55] 2021 – *Systematic Review*	66	India	Maternal & Child Health – *investigating the impact of public health programs on maternal and child health*	Trained female health workers (ASHAs) serve as the interface between communities and health facilities and spread awareness on health	ANC (1)	*This review presented one study with ASHAs which demonstrated an increase in ANC.*
Takah et al [52] 2019 – *Systematic Review & Meta-Analysis*	8	Sub-Saharan Africa	PMTCT of HIV – *examining interventions used to improve male partner involvement in PMTCT*	Community meetings (talks, music, dance, dramas)	ANC (1)	*This review did not present outcomes related to ANC/PNC care-seeking.*
Yuan et al [54] 2014 – *Systematic Review*	22	LMICs	Maternal & Child Health – *collecting evidence on the effects of interventions to reduce maternal and child health inequalities*	Participatory women's group	ANC (2)	One intervention in Bangladesh demonstrated a reduction in income inequalities in access to ANC.

LMIC – low and middle-income country, ANC – antenatal care, PNC – postnatal care, PLA – participatory learning and action, HIV – human immunodeficiency virus, PMTCT – prevention of mother-to-child-transmission

*The summary table is organized in two parts – the first half are the 12 reviews that contained a significant level of detail describing the community mobilization that could be synthesized in the rest of this review of reviews, the second half of this table displays the 11 reviews that met inclusion criteria but did not provide additional levels of detail. The information presented in the table reflects what was reported in the included review and does not go into depth of the primary studies included in the reviews.

Most reviews focused broadly on RMNCH [21,23,36,38-40,42-44,46-51,53,54]. One review specifically focused on ANC [45], one on PNC [37], and one on both ANC and PNC [41]. The last two included reviews focused on maternal health for women with HIV or prevention of mother-to-child-transmission (PMTCT) of HIV [20,52]. Eleven of the reviews aimed to synthesize evidence on community mobilization and/or community-based interventions and MNH outcomes [20,21,23,36,42,44,45,47-51]. The reviews varied in which MNH outcomes were investigated, especially in relation to community mobilization. The majority of the reviews used only ANC uptake (n = 17), including three reviews that reported on early ANC [39,42,45,55]. The remaining reviews looked at ANC and PNC outcomes for the community mobilization interventions (n = 4) or only PNC (n = 1).

### Findings of community mobilization on ANC/PNC uptake

Overall, reviews describe a positive association between community mobilization and ANC/PNC uptake. The ANC/PNC utilization outcomes documented by the reviews varied, perhaps explaining the dearth of meta-analyses conducted. Reviews described specific outcomes such as number of antenatal examinations [39], utilizing antenatal health facilities [44], or receiving at least one ANC visit [39,42,45,51]. Three of these reviews also investigated receiving at least three or four ANC visits [39,42,45]. The five reviews that stated PNC-specific outcomes described awareness of PNC [37] and postpartum care-seeking [21] or PNC check-ups for the mother and/or newborn [41,49,53]. The three reviews with studies that examined ANC in the first trimester reported on registration [39,42] and actual attendance [45]. Detailed findings of the reviews are presented in Appendix S4 in the **Online Supplementary Document**.

While it was not possible to synthesize results in meta-analyses due to high heterogeneity across included studies, 15 reviews reported at least one positive association between what they described as community mobilization activities and ANC and/or PNC uptake [20,21,23,36,38-40,42-45,47,48,51,53,54]. However, five reviews also presented primary studies that found no change or no statistically significant changes in ANC uptake despite the implementation of community mobilization interventions [21,39,42,45,47] and one referenced a study that found a decrease in the “overall use of antenatal facilities” [44]. Though more limited, the findings on PNC uptake were also largely positive with two reviews describing significant increases in women attending PNC following the interventions [49,53].

### Classification of community mobilization strategies to increase community support for ANC/PNC uptake

The varying levels of detail presented in the reviews presented challenges in synthesizing findings across all reviews. The 12 reviews (13 papers) that described community mobilization and ANC/PNC in greater depth comprise the main focus for the rest of this publication. As demonstrated in **Table 3**, the reviews included in this analysis had a range of different stated objectives and the primary studies they synthesized would have also differed in their objectives and studied interventions. Four of the reviews focused on assessing interventions including community mobilization and/or participation [21,42,44,51], while the rest of the reviews did not specify a focus on community mobilization or considered community-based interventions more broadly.

Additionally, there are various definitions and levels of community mobilization; and an array of activities that are called community mobilization. This review of reviews highlights that heterogeneity as well. Strategies and activities described by the reviews ranged from passive communication to more active approaches that included involving community members or consulting local leaders. **Table 4** displays the range of activities presented in the reviews as community mobilization strategies. The majority of included reviews did not go into depth on how these activities were implemented, such as the topics covered in meetings or the process of developing communication materials.

**Table 4:** Community mobilization strategies for ANC/PNC described in the included reviews with significant level of detail (n = 12)

Range/Type/Level of Mobilization	Type of activity	Description of activities	References
*These activities place a focus on problem solving and finding solutions.*	**Community health committees**	Community health committees were set up as an opportunity to consult various community members, collaborate, build consensus, and identify solutions to maternal and newborn health problems.	Kuhlmann et al [42], Jennings et al [47] & Perry et al [48]
	**Local leader meetings**	Meetings with local leaders could be used in various forms at different time points in an intervention. Some interventions engaged with local leaders at the start for buy-in/involvement in initiatives such as women's groups or other community activities while others had elected local leaders run community meetings with the public and stakeholders.	George et al [39], Kearns et al [41], Perry et al [47,48]
	**Recurrent groups**	These activities included groups which met on a regular or recurring basis to discuss and address maternal health issues. This included women’s groups led by volunteers as well as groups using the PLA cycle where a trained facilitator led regularly scheduled meetings. These recurrent groups often utilized participatory activities that were used to identify and adopt strategies in the community to improve maternal health.	George et al [39], Kearns et al [41], Marston et al [44], Mbuagbaw et al [45], Mangham-Jeffries et al [43], Perry et al [47,48], Prost et al [21]
*These activities involve participatory techniques to mobilize communities for immediate action.*	**Peer mentors**	Peer mentors or peer counsellors were used to provide education, advice, and support to pregnant women and families. They often used participatory learning activities or community dialogues to encourage community action. One example of peer mentors called “Care Groups” used facilitators to share health education that volunteer participants could then disseminate to mothers in their surrounding households[48]	Kuhlmann et al [42], Mangham-Jeffries et al [43], Perry et al [47,48]
	**Public/ community meetings**	Larger community gatherings where trained volunteers or health care workers provided information and education as well as identified community action plans and priorities. Various strategies and activities were employed at these community awareness meetings including: street plays, dramas, dances, music, folksongs, skits, games, and other participatory learning activities and methods.	George et al [39], Mbuagbaw et al [45], Perry et al [47,48], Sarkar et al [49], Sharma et al [51]
	**Advocacy workshops & special community events**	Public education and advocacy activities were often held to increase demand for maternal health services. This included special community events such as health fairs and celebration days to promote awareness and encourage community support for health interventions. For example, one study reported women's groups encouraging attendance at “Mamta Divas” which were special event village health and nutrition days for mothers and children[39].	George et al [39], Sarkar et al [49], Sharma et al [51]
*These activities involve informing or educating communities.*	**Mass media & awareness campaigns**	Mass media and awareness campaigns were conducted through media forums such as radio, television, newspapers, cellular phone messages and printed materials such as posters, brochures, and banners as well as live events such as street theatre. The aim of these interventions was often to inform the community and pregnant women in order to improve health service utilization, such as ANC and PNC.	George et al, Lassi et al, Mbuagbaw et al [45]
	**Women & men's maternal health education sessions**	Community mobilisers, health care workers, and midwives held focus groups with community members or visited households to discuss and educate both men and women on maternal health. These activities were conducted by both men and women community mobilisers. Also defined as “group counselling.”[23]	Lassi et al [23], Mangham-Jeffries et al [43], Sharma et al [51]
	**Home visits+ (as a component of larger intervention package)**	While home visits alone are not typically considered a community mobilization activity on their own, many of the reviews and studies described home visits as a component of community-based interventions and one of the objectives of the visit was to mobilize family support for MNH. Sessions aimed used to provide interactive education as well as support for care seeking for health services as well as improve household care practices. These were conducted by community health workers, community organizers, or peer counsellors. Also defined as “one-to-one counselling.” [23]	George et al [39], Kuhlmann et al [42], Lassi et al [23], Mangham-Jeffries et al [43], Mbuagbaw et al [45], Perry et al [47,48], Wekesah et al [53]

ANC – antenatal care, PNC – postnatal care, PLA – participatory and learning action, MNH – maternal and newborn health

### Community mobilization strategies to increase community support for early ANC

Three reviews included one study each that identified early use of ANC as an outcome. The findings and strategies presented by the relevant primary studies are summarized below. George et al. described a study using discussions and participatory exercises in women’s groups to develop a monitoring tool for maternal health care [39]. The tool was shared with local leaders and stakeholders to develop plans of action to improve the quality of maternal health services [57]. ANC registration in the first trimester increased in both communities where this programme was implemented – from 31.4% to 54.3% in Dhabva and from 17% to 41.8% in Sevaniya [57]. Mbuagbaw et al. reported on a study by Wu et al. that used a multipart intervention to train midwives, inform communities on ANC, and provide basic medical resources to improve ANC and therefore MNH outcomes [45,58]. The trained midwives distributed print materials such as letters, leaflets, and educational posters promoting ANC [58]. However, in combination with the other components of the intervention, this program did not improve uptake of ANC earlier in pregnancy [45]. The final study included in Kuhlman et al. focused on community-based interventions to conduct trainings for community members and birth attendants led by government officials and local community leaders [59]. While the primary focus of the study was on newborn mortality, they also reported an increase in scheduled ANC visits for primigravida women in the first 16 weeks of pregnancy (from 18.75% to 56.9%, *P* < 0.001) [42].

### Factors influencing ANC uptake and community mobilization

**Figure 2:**
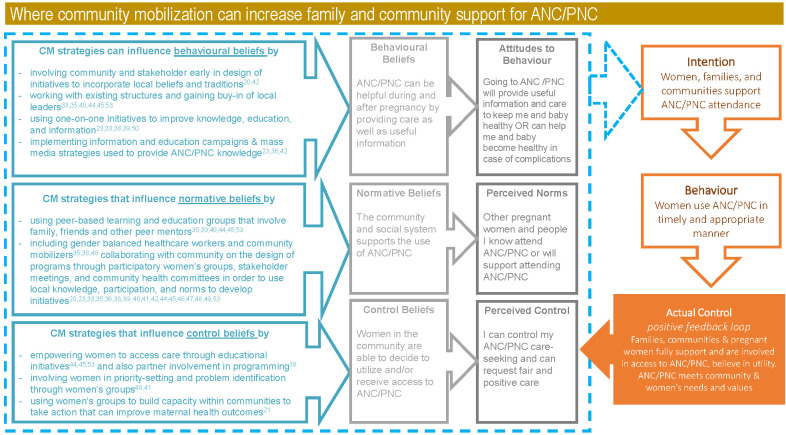
Logic model mapping. The logic model includes “What do people believe in this context (behavioral beliefs)?'; 'What is normal in this context' (normative beliefs)? and 'How much control do we have over what happens here' (control beliefs)?; the attitudes and perceptions predicted by these beliefs; the intended behavior that could result; and the actual experiences” [27] and cyclical feedback loop that connects all of these components.

A recent qualitative evidence synthesis on uptake of routine ANC, conducted to inform the development of WHO’s ANC recommendations, identified 52 factors (perceptions or experiences of women and health care providers) across three overarching domains (behavioral beliefs, normative beliefs, and control beliefs) that influenced the initial or continued use of ANC [27]. Appendix S5 in the **Online Supplementary Document** exhibits how 25 of these factors can be influenced by or supported with community mobilization based on insights from the included reviews. Using the evidence, the synthesis developed a logic model based on the theory of planned behavior to map how individual and social beliefs and norms influenced women’s decisions to attend ANC regularly [27,34]. This model depicts how background factors that extend beyond the individual, influence women’s beliefs, attitudes, and finally behaviours regarding ANC (and potentially PNC) [11,27]. In order to provide more pragmatic insight from the findings of this review of reviews, we considered where community mobilization can influence the pathway described by the positive feedback loop logic model. **Figure 2** illustrates what components and strategies from community mobilization activities can influence behavioral beliefs, normative beliefs, and control beliefs which would increase community support for ANC/PNC uptake.

Behavioral beliefs may be influenced by the information and knowledge individuals have. Community mobilization strategies that focus on information, education, communication, and awareness can influence these initial behavioral beliefs by providing additional information. However, reviews also highlight the importance of incorporating local knowledge and tradition into these methods and communication materials [20,45]. Normative beliefs are swayed by social and community influences, highlighting the relevance of community mobilization strategies that target not only pregnant and postpartum women but also the partner, their family and friends. Societal norms such as power, family dynamics, and gender-balances must be considered in the program design [38,41,51]. Participatory methodologies that include women, parents, local leaders, and community stakeholders can incorporate local knowledge and norms in the development of initiatives [20,23,36,38,39,41-45,47-52]. Beliefs about the level of control individuals have are where empowerment of individuals, and particularly pregnant and postpartum women and parents, becomes more impactful. This stream goes beyond the involvement and consultation of pregnant women. Parents and the community by a particular program to a sustainable and continued capacity for community members to identify and participate in addressing challenges. A range of community mobilization activities can influence control beliefs to empower women and parents beginning with education to establishing women’s groups that focus on maternal and child health activities [21].

## DISCUSSION

The objective of this review of reviews was to identify community mobilization strategies used to support appropriate and timely use of ANC (including early access in the first trimester) and PNC in LMICs. This review of reviews identified 23 publications representing 22 reviews that described using community mobilization to influence ANC/PNC uptake. The reviews ranged in their methods, objectives, and descriptions of community mobilization strategies as well as their definitions of early ANC (12 vs 16 weeks). Overall, the majority of studies noted the importance of community mobilization in delivering or improving support for ANC/PNC uptake either as a main intervention or within a package of interventions. However, reviews largely focused on the statistical outcomes of including a “community mobilization component” such as changes in uptake of services or mortality rates. While many of these reviews stated or implied an element of community mobilization was present, they often did not describe in sufficient detail what this process entailed or how it was implemented [19]. As a result, the majority of reported findings in the literature highlight community mobilization’s positive influence on ANC/PNC use, but lack information on the processes to explain this association. This review of reviews details positive findings relating to community mobilization to improve family and community support for ANC/PNC, but it is difficult to make more specific recommendations due to little insight into how or why these approaches work.

The logic model presented by the Downe et al. qualitative evidence synthesis and adapted in **Figure 2** provides a lens to understand what is happening between the external intervention and the final outcome of using ANC/PNC. Community mobilization activities can influence this pathway through targeted interventions that influence behavioral, normative, and control beliefs. Information-education-communication (IEC) approaches target behavioral beliefs by using public health education or communication to affect behavior change in a population [60]. In line with a range of community-related strategies, it is important to incorporate community norms and beliefs and work with community leaders in the development and implementation of IEC [60]. One example rolled out for child health in South Asia is the UNICEF Meena Communication Platform that used the animated character of “Meena”, a young South Asian girl, to communicate important health messaging in an entertaining and relatable format [46,61]. Adapting community mobilization strategies through the involvement of local stakeholders in the design process enables the contextualization of materials and programming [20,38,45].

This involvement of community members can extend to the inclusion of women and families through participatory activities and peer groups that are implemented in ways that respect societal norms. A methodology described by multiple reviews is the Warmi methodology, first introduced by Save the Children in Bolivia in 1990 [62]. This multi-step PLA cycle used in women’s groups not only provided beneficial peer support, but also empowered women to make decisions and take action to implement local solutions for the challenges they faced in regards to their and their baby’s health [21,38,63]. Through the Warmi project, women played an active role in planning how their own priorities would be addressed by developing educational materials and strategies alongside local authorities, as well as continued involvement of the community and women through participatory evaluation practices [40,44,64,65]. Community health committees also use a collaborative approach to increase local programme ownership and therefore community empowerment [66]. By working directly with local communities, initiatives to improve community support for ANC/PNC place power and responsibility in these communities. This belief and perception of control plays a role in the uptake logic model feedback loop both on an individual and community level [27,34].

These findings have implications for practice, policy, and research. As established in the literature, a range of factors influence the utilization of appropriate and timely ANC/PNC [7,8,11,27,67-69]. Often these are community-related factors which have supported the use of community mobilization to enable community action and contribute to improved MNH [70-72]. The adapted logic model demonstrates opportunities for community mobilization strategies to influence the perceptions and experiences of communities in regards to support for ANC/PNC [27]. As highlighted by this review of reviews, a range of activities and programs have been implemented across scales and settings. However, the complexity of these interventions as well as the lack of information on implementation processes advocate for further studies that provide this additional detail on community mobilization’s implications for ANC/PNC support and uptake. Reviews published as recently as 2021 perpetuate the same pattern by labeling one-sided didactic programs as “community participation” or failing to describe what comprised the community mobilization component of a program [73,74]. Furthermore, while the reviews show a large geographic range in terms of countries and SDG regions included, the relevance of context in community-based interventions emphasizes the importance of considering localized and country/region-specific findings. This is especially important when applying lessons learned from global trends across settings.

Notably, barriers and facilitators are different across contexts making these programs a complex health intervention which could benefit from being considered through an implementation science or complexity science lens [75]. Implementation science methodologies could provide a systems-level consideration of the processes of community mobilization, rather than a positivist approach reporting on binary outcomes [76,77]. In order to learn from the experiences of community mobilization strategies and implement them in new or future settings, there is a need to understand generative causation and how these programs work for individuals and families within social systems [78]. In order to have a sustainable impact, community mobilization inherently relies on varying levels of systems as well as inter-related decision-making and behaviours of individuals and groups that form dynamic feedback loops and dependencies [13,76,79]. This implementation science lens can be used to account for the dynamic nature of these interventions to inform the translation of findings into recommendations for policy and practice [76,77].

### Strengths and limitations

By analyzing literature at the review level, this paper provides a comprehensive overview of the existing evidence and highlights implications for policy and practice as well as research gaps. Notably, this review of reviews emphasizes how the current descriptions of incorporating these community mobilization activities are often limited in detail or a brief sentence in a publication’s methodology section. There is a need to document and learn from the processes of community mobilization strategies when they are implemented, often within larger MNH packages. However, in order to do this and guide eventual implementation this review of reviews also calls for programmers and researchers to clearly define and explain the strategies and processes used to mobilize a community. **Table 4** provided above may serve as a starting point.

The high heterogeneity of review designs made it challenging to synthesize findings across a varied range of interventions and outcomes. This heterogeneity comes from the variety of review objectives, activities, and reported metrics and outcomes. The included reviews presented varying levels of detail in their findings – some reviews reported specific study results, while others alluded to groups of studies more generally. Additionally, as this is a review of reviews, some primary studies were represented in multiple reviews which may have emphasized or over-represented some individual studies’ findings. Finally, ANC and PNC were often not the main focus of the reviews. Many reviews considered sexual and reproductive or maternal, newborn, and child health more broadly with ANC/PNC as one component or outcome. As a result, increasing community support for ANC/PNC specifically was often not the main focus of the interventions and strategies they described. This implies community mobilization activities described in this review cannot be simply extrapolated to improve ANC/PNC uptake, but they will need to be part of broader MNH programme strategy as demonstrated by many of the included studies [17,18].

## A call for action and clarity

Since the Alma Ata Declaration that prioritized community participation in health care, programs and policies that include community members in all aspects of health care have been widely advocated for within MNH [80]. Community mobilization approaches are amongst these strategies and are designed to give the community an opportunity to contribute to improved MNH and increase community support for appropriate and timely use of ANC and PNC and contribute to empowering women, parents and communities. These actions are not just an add-on to health interventions but is a right that must be unequivocally integrated into any initiative. Additionally, the range of community mobilization strategies can also shape the behavioral, normative, and control beliefs of both individuals and communities – influencing the pathways in the logic model for uptake of timely and appropriate ANC/PNC.

Community-based interventions, and particularly community mobilization approaches are emphasized in MNH programmes throughout different global strategies. Yet, this review affirms that there is little consistency in what this means, how it is implemented and poor documentation that allows us to understand and appreciate what has been done and what was the effect [81]. As noted in previous reviews and the literature, there is ambiguity surrounding the various definitions and what qualifies as “community mobilization,” and there is often overlap with additional terms such as “community engagement,” “community consultation,” or “community participation” [20,81,82]. These differing terminologies reflect the complex and dynamic nature of “community–(engagement/mobilization/participation)” [81-83]. These findings therefore calls for those in the field to harmonize definitions of this work, and to more robustly document and report the operationalization and processes by using reporting standards such as the WHO Programme Reporting Standards for sexual, reproductive, maternal, newborn, child and adolescent health [84]. This documentation can then guide and support evidence-informed implementation of community strategies for ANC/PNC as well as wider maternal and child health.

## Additional material


Online Supplementary Document
