# Supplementary material for: Community mobilization to strengthen support for appropriate and timely use of antenatal and postnatal care: A review of reviews
Source: J Glob Health. 2021 Dec 30;11:04076. doi: 10.7189/jogh.11.04076 (PMC8710228; doi:10.7189/jogh.11.04076)
Supplement: Online Supplementary Document [file jogh-11-04076-s001.pdf]

Supplementary Material

Table of Contents

Appendix 1: Search strategy *2*

Appendix 2: JBI Critical Appraisal *9*

Appendix 3: Sustainable Development Goals (SDG) regions represented in reviews *11*

Appendix 4: Key findings as reported by reviews *13*

Appendix 5: Additional evidence reported in reviews to support Downe et al. qualitative evidence synthesis *24*

Appendix 1: Search strategy

1	ANC/PNC<br><br>TITLE	(antenatal OR prenatal OR pregnan* OR matern* OR “child health” OR postnatal OR perinatal OR reproductive OR birth OR “family plan*” OR infant* OR mother* OR women OR woman OR ANC OR PNC)
2	Community Mobilization Strategies<br><br>TITLE/ABSTRACT	(communit* OR public OR stakeholder OR local* OR social OR patient* OR consumer*)<br><br>NEAR/2<br><br>(mobiliz* OR mobilis* OR engage* OR participat* OR sensiti* OR communicat* OR messag* OR dialog* OR outreach OR promot* OR media OR capacity OR educat* OR interven* OR behavio*)
3	Reviews<br><br>TITLE	review OR systematic OR synthes*
4	Demand<br><br>TITLE	utilis* OR utiliz* OR promot* OR uptake

1 AND 2 AND 3
1 AND 3 AND 4

CINAHL – 28 JAN – 374 RESULTS

S1 TI antenatal OR prenatal OR pregnan* OR matern* OR “child health” OR postnatal OR perinatal OR reproductive OR birth OR “family plan*” OR infant* OR mother* OR women OR woman OR ANC OR PNC

S2 TI review OR systematic OR synthes*

S3 TI utilis* OR utiliz* OR promot* OR uptake

S4 TI (communit* OR public OR stakeholder OR local* OR social OR patient* OR consumer*) N2 (mobiliz* OR mobilis* OR engage* OR participat* OR sensiti* OR communicat* OR messag* OR dialog* OR outreach OR promot* OR media OR capacity OR educat* OR interven* OR behavio*)

S5 AB (communit* OR public OR stakeholder OR local* OR social OR patient* OR consumer*) N2 (mobiliz* OR mobilis* OR engage* OR participat* OR sensiti* OR communicat* OR messag* OR dialog* OR outreach OR promot* OR media OR capacity OR educat* OR interven* OR behavio*)

S6 S1 AND S2 AND S3

S7 S4 OR S5

S8 S1 AND S2 AND S7

S9 S6 OR S8

PUBMED – 28 JAN – 235 RESULTS

("review"[Title] OR "systematic"[Title] OR "synthes*"[Title]) AND ("antenatal"[Title] OR "prenatal"[Title] OR "pregnan*"[Title] OR "matern*"[Title] OR "child health"[Title] OR "postnatal"[Title] OR "perinatal"[Title] OR "reproductive"[Title] OR "birth"[Title] OR "family plan*"[Title] OR "infant*"[Title] OR "mother*"[Title] OR "women"[Title] OR "woman"[Title] OR "ANC"[Title] OR "PNC"[Title]) AND ((("communit*"[Title/Abstract] OR "public"[Title/Abstract] OR "stakeholder"[Title/Abstract] OR "local*"[Title/Abstract] OR "social"[Title/Abstract] OR "patient*"[Title/Abstract] OR "consumer*"[Title/Abstract]) N2 ("mobiliz*"[Title/Abstract] OR "mobilis*"[Title/Abstract] OR "engage*"[Title/Abstract] OR "participat*"[Title/Abstract] OR "sensiti*"[Title/Abstract] OR "communicat*"[Title/Abstract] OR "messag*"[Title/Abstract] OR "dialog*"[Title/Abstract] OR "outreach"[Title/Abstract] OR "promot*"[Title/Abstract] OR "media"[Title/Abstract] OR "capacity"[Title/Abstract] OR "educat*"[Title/Abstract] OR "interven*"[Title/Abstract] OR "behavio*"[Title/Abstract]))) OR (("review"[Title] OR "systematic"[Title] OR "synthes*"[Title]) AND ("antenatal"[Title] OR "prenatal"[Title] OR "pregnan*"[Title] OR "matern*"[Title] OR "child health"[Title] OR "postnatal"[Title] OR "perinatal"[Title] OR "reproductive"[Title] OR "birth"[Title] OR "family plan*"[Title] OR "infant*"[Title] OR "mother*"[Title] OR "women"[Title] OR "woman"[Title] OR "ANC"[Title] OR "PNC"[Title]) AND ("utilis*"[Title] OR "utiliz*"[Title] OR "promot*"[Title] OR "uptake"[Title]))

PSYCINFO – 28 JAN – 341 RESULTS – WITH DEMAND WORDS

(ti(review OR systematic OR synthes*) AND ti((antenatal OR prenatal OR pregnan* OR matern* OR "child health" OR postnatal OR perinatal OR reproductive OR birth OR "family plan*" OR infant* OR mother* OR women OR woman OR ANC OR PNC)) AND ti(utilis* OR utiliz* OR promot* OR uptake)) OR (ti(review OR systematic OR synthes*) AND ti((antenatal OR prenatal OR pregnan* OR matern* OR "child health" OR postnatal OR perinatal OR reproductive OR birth OR "family plan*" OR infant* OR mother* OR women OR woman OR ANC OR PNC)) AND noft((communit* OR public OR stakeholder OR local* OR social OR patient* OR consumer*) NEAR/2 (mobiliz* OR mobilis* OR engage* OR participat* OR sensiti* OR communicat* OR messag* OR dialog* OR outreach OR promot* OR media OR capacity OR educat* OR interven* OR behavio*)))

EMBASE – 28 JAN – 662 RESULTS – WITH DEMAND WORDS

Query((antenatal:ti OR prenatal:ti OR pregnan*:ti OR matern*:ti OR 'child health':ti OR postnatal:ti OR perinatal:ti OR reproductive:ti OR birth:ti OR 'family plan*':ti OR infant*:ti OR mother*:ti OR women:ti OR woman:ti OR anc:ti OR pnc:ti) AND (review:ti OR systematic:ti OR synthes*:ti) AND (utilis*:ti OR utiliz*:ti OR promot*:ti OR uptake:ti) OR ((antenatal:ti OR prenatal:ti OR pregnan*:ti OR matern*:ti OR 'child health':ti OR postnatal:ti OR perinatal:ti OR reproductive:ti OR birth:ti OR 'family plan*':ti OR infant*:ti OR mother*:ti OR women:ti OR woman:ti OR anc:ti OR pnc:ti) AND (review:ti OR systematic:ti OR synthes*:ti) AND (((communit* OR public OR stakeholder OR local* OR social OR patient* OR consumer*) NEAR/2 (mobiliz* OR mobilis* OR engage* OR participat* OR sensiti* OR communicat* OR messag* OR dialog* OR outreach OR promot* OR media OR capacity OR educat* OR interven* OR behavio*)):ab,ti))) AND [2000-2021]/py

COCHRANE – 28 JAN – 32 RESULTS – WITH DEMAND WORDS

Search Name:
Date Run: 28/01/2021 23:14:38
Comment:

ID Search Hits
#1 (review OR systematic OR synthes*):ti (Word variations have been searched) 10311
#2 (utilis* OR utiliz* OR promot* OR uptake):ti (Word variations have been searched) 13491
#3 ((communit* OR public OR stakeholder OR local* OR social OR patient* OR consumer*) NEAR/2 (mobiliz* OR mobilis* OR engage* OR participat* OR sensiti* OR communicat* OR messag* OR dialog* OR outreach OR promot* OR media OR capacity OR educat* OR interven* OR behavio*)):ti,ab,kw (Word variations have been searched) 75251
#4 (antenatal OR prenatal OR pregnan* OR matern* OR "child health" OR postnatal OR perinatal OR reproductive OR birth OR "family plan*" OR infant* OR mother* OR women OR woman OR ANC OR PNC):ti91867
#5 #1 AND #2 AND #4 9
#6 #1 AND #3 AND #4 26
#7 #5 OR #6 34

PROSPERO – 28 JAN – 133 RESULTS – WITH DEMAND WORDS
#1 (antenatal OR prenatal OR pregnan* OR matern* OR child health OR postnatal OR perinatal OR reproductive OR birth OR family plan* OR infant* OR mother* OR women OR woman OR ANC OR PNC):TI 6550

#2 (review OR systematic OR synthes*):TI 76685

#3 (utilis* OR utiliz* OR promot* OR uptake):TI 1230

#4 ((communit* OR public OR stakeholder OR local* OR social OR patient* OR consumer*) NEAR2 (mobiliz* OR mobilis* OR engage* OR participat* OR sensiti* OR communicat* OR messag* OR dialog* OR outreach OR promot* OR media OR capacity OR educat* OR interven* OR behavio*)):TI 579

#5 #2 AND #3 AND #1 123

#6 #1 AND #2 AND #4 12

#7 #5 OR #6 133

MASCOT – 28 JAN – 20 RESULTS – WITH DEMAND WORDS

Search | Search history | Studies

List of items retrieved in the selected search(s)

The table below contains the studies retrieved by your search. Click 'Select' to view the study details and manually assigned keywords.
Your search terms were: *62 OR 63*

Showing records 1 to 20 of 20

Study	Title	View details
Author (0)	Pain and women's satisfaction with the experience of childbirth: a systematic review	Select
Bhutta (2005)	Community-based interventions for improving perinatal and neonatal health outcomes in developing countries: a review of the evidence.	Select
Bigirwa (2009)	Effectiveness of community health workers (CHWS) in the provision of basic preventive and curative maternal, newborn and child health (MNCH) interventions: A systematic review.	Select
Brusse (2010)	Electroencephalography during normotensive and hypertensive pregnancy: A systematic review	Select
Car (2012)	Integrating prevention of mother-to-child HIV transmission programs to improve uptake: A systematic review	Select
de Oliveira (2001)	Extending breastfeeding duration through primary care: a systematic review of prenatal and postnatal interventions.	Select
Kennedy (2010)	Linking sexual and reproductive health and HIV interventions: a systematic review	Select
Mantel (2002)	Can a developed country's maternal mortality review be used as the 'gold standard' for a developing country?	Select
Marston (2013)	Effects of Community Participation on Improving Uptake of Skilled Care for Maternal and Newborn Health: A Systematic Review.	Select
McMahan (2001)	India: Community Partnerships for Safe Motherhood (CPSM). Empowered women and families: promoting better health behaviors. Results review.	Select
Murray (2012)	Demand-side financing measures to increase maternal health service utilisation and improve health outcomes: a systematic review of evidence from low- and middle-income countries	Select
Nyamtema (2011)	Maternal health interventions in resource limited countries: a systematic review of packages, impacts and factors for change	Select
Pai (2007)	Rapid point-of-care HIV testing in pregnant women: a systematic review and meta-analysis	Select
Pell (2011)	Social and cultural factors affecting uptake of interventions for malaria in pregnancy in Africa: a systematic review of the qualitative research.	Select
Prost (2013)	Women's groups practising participatory learning and action to improve maternal and newborn health in low-resource settings: a systematic review and meta-analysis	Select
Rojahn (2011)	Community-Based Intervention Packages for Reducing Maternal and Neonatal Morbidity and Mortality and Improving Neonatal Outcomes: A Review Synopsis.	Select
Say (2007)	A systematic review of inequalities in the use of maternal health care in developing countries: Examining the scale of the problem and the importance of context.	Select
Thangaratinam (2012)	Interventions to reduce or prevent obesity in pregnant women: A systematic review	Select
Vieira (2012)	Increasing the Use of Skilled Health Personnel Where Traditional Birth Attendants Were Providers of Childbirth Care: A Systematic Review	Select
Worrall (2007)	The economics of malaria in pregnancy--a review of the evidence and research priorities.	Select

(Landscape mode works best if you are printing wide search results or report tables.)

TOTAL IN COVIDENCE (without MASCOT) AFTER DUPLICATES = 1150

2 SEPTEMBER 2021: searching for publications from Jan 2021

CINAHL – 2 SEP – 4 RESULTS

S3	(TI (communit* OR public OR stakeholder OR local* OR social OR patient* OR consumer*) N2 (mobiliz* OR mobilis* OR engage* OR participat* OR sensiti* OR communicat* OR messag* OR dialog* OR outreach OR promot* OR media OR capacity OR educat* OR interven* OR behavio*) OR AB (communit* OR public OR stakeholder OR local* OR social OR patient* OR consumer*) N2 (mobiliz* OR mobilis* OR engage* OR participat* OR sensiti* OR communicat* OR messag* OR dialog* OR outreach OR promot* OR media OR capac ...	View Results (4) View Details Edit
S2	TI ((communit* OR public OR stakeholder OR local* OR social OR patient* OR consumer*) N2 (mobiliz* OR mobilis* OR engage* OR participat* OR sensiti* OR communicat* OR messag* OR dialog* OR outreach OR promot* OR media OR capacity OR educat* OR interven* OR behavio*)) OR AB ((communit* OR public OR stakeholder OR local* OR social OR patient* OR consumer*) N2 (mobiliz* OR mobilis* OR engage* OR participat* OR sensiti* OR communicat* OR messag* OR dialog* OR outreach OR promot* OR media OR ...	View Results (194,695) View Details Edit
S1	TI (antenatal OR prenatal OR pregnan* OR matern* OR “child health” OR postnatal OR perinatal OR reproductive OR birth OR “family plan*” OR infant* OR mother* OR women OR woman OR ANC OR PNC) AND TI (review OR systematic OR synthes*) AND TI (utilis* OR utiliz* OR promot* OR uptake)	View Results (152) View Details Edit

PUBMED – 2 SEP – 38 RESULTS

("review"[Title] OR "systematic"[Title] OR "synthes*"[Title]) AND ("antenatal"[Title] OR "prenatal"[Title] OR "pregnan*"[Title] OR "matern*"[Title] OR "child health"[Title] OR "postnatal"[Title] OR "perinatal"[Title] OR "reproductive"[Title] OR "birth"[Title] OR "family plan*"[Title] OR "infant*"[Title] OR "mother*"[Title] OR "women"[Title] OR "woman"[Title] OR "ANC"[Title] OR "PNC"[Title]) AND ((("communit*"[Title/Abstract] OR "public"[Title/Abstract] OR "stakeholder"[Title/Abstract] OR "local*"[Title/Abstract] OR "social"[Title/Abstract] OR "patient*"[Title/Abstract] OR "consumer*"[Title/Abstract]) N2 ("mobiliz*"[Title/Abstract] OR "mobilis*"[Title/Abstract] OR "engage*"[Title/Abstract] OR "participat*"[Title/Abstract] OR "sensiti*"[Title/Abstract] OR "communicat*"[Title/Abstract] OR "messag*"[Title/Abstract] OR "dialog*"[Title/Abstract] OR "outreach"[Title/Abstract] OR "promot*"[Title/Abstract] OR "media"[Title/Abstract] OR "capacity"[Title/Abstract] OR "educat*"[Title/Abstract] OR "interven*"[Title/Abstract] OR "behavio*"[Title/Abstract]))) OR (("review"[Title] OR "systematic"[Title] OR "synthes*"[Title]) AND ("antenatal"[Title] OR "prenatal"[Title] OR "pregnan*"[Title] OR "matern*"[Title] OR "child health"[Title] OR "postnatal"[Title] OR "perinatal"[Title] OR "reproductive"[Title] OR "birth"[Title] OR "family plan*"[Title] OR "infant*"[Title] OR "mother*"[Title] OR "women"[Title] OR "woman"[Title]

OR "ANC"[Title] OR "PNC"[Title]) AND ("utilis*"[Title] OR "utiliz*"[Title] OR "promot*"[Title] OR "uptake"[Title]))

PSYCHINFO – 2 SEP – 18 RESULTS

(ti(review OR systematic OR synthes*) AND ti((antenatal OR prenatal OR pregnan* OR matern* OR "child health" OR postnatal OR perinatal OR reproductive OR birth OR "family plan*" OR infant* OR mother* OR women OR woman OR ANC OR PNC)) AND ti(utilis* OR utiliz* OR promot* OR uptake)) OR (ti(review OR systematic OR synthes*) AND ti((antenatal OR prenatal OR pregnan* OR matern* OR "child health" OR postnatal OR perinatal OR reproductive OR birth OR "family plan*" OR infant* OR mother* OR women OR woman OR ANC OR PNC)) AND noft((communit* OR public OR stakeholder OR local* OR social OR patient* OR consumer*) NEAR/2 (mobiliz* OR mobilis* OR engage* OR participat* OR sensiti* OR communicat* OR messag* OR dialog* OR outreach OR promot* OR media OR capacity OR educat* OR interven* OR behavio*)))

EMBASE – 2 SEP – 89 RESULTS

#1 AND **2021**:py

89

#1 (**antenatal**:ti OR **prenatal**:ti OR **pregnan***:ti OR **matern***:ti OR **'child health'**:ti OR **postnatal**:ti OR **perinatal**:ti OR **reproductive**:ti OR **birth**:ti OR **'family plan*'**:ti OR **infant***:ti OR **mother***:ti OR **women**:ti OR **woman**:ti OR **anc**:ti OR **pnc**:ti) AND (**review**:ti OR **systematic**:ti OR **synthes***:ti) AND (**utilis***:ti OR **utiliz***:ti OR **promot***:ti OR **uptake**:ti) OR ((**antenatal**:ti OR **prenatal**:ti OR **pregnan***:ti OR **matern***:ti OR **'child health'**:ti OR **postnatal**:ti OR **perinatal**:ti OR **reproductive**:ti OR **birth**:ti OR **'family plan*'**:ti OR **infant***:ti OR **mother***:ti OR **women**:ti OR **woman**:ti OR **anc**:ti OR **pnc**:ti) AND (**review**:ti OR **systematic**:ti OR **synthes***:ti) AND (((**communit*** OR **public** OR **stakeholder** OR **local*** OR **social** OR **patient*** OR **consumer***) NEAR/2 (**mobiliz*** OR **mobilis*** OR **engage*** OR **participat*** OR **sensiti*** OR **communicat*** OR **messag*** OR **dialog*** OR **outreach** OR **promot*** OR **media** OR **capacity** OR **educat*** OR **interven*** OR **behavio***)):ab,ti))

772

COCHRANE – 2 SEP – 0 RESULTS

ID	Search	Hits
#1	(review OR systematic OR synthes*):ti (Word variations have been searched)	10505
#2	(utilis* OR utiliz* OR promot* OR uptake):ti (Word variations have been searched)	14371
#3	((communit* OR public OR stakeholder OR local* OR social OR patient* OR consumer*) NEAR/2 (mobiliz* OR mobilis* OR engage* OR participat* OR sensiti* OR communicat* OR messag* OR dialog* OR outreach OR promot* OR media OR capacity OR educat* OR interven* OR behavio*)):ti,ab,kw (Word variations have been searched)	79459
#4	(antenatal OR prenatal OR pregnan* OR matern* OR “child health” OR postnatal OR perinatal OR reproductive OR birth OR “family plan*” OR infant* OR mother* OR women OR woman OR ANC OR PNC):ti	96300
#5	#1 AND #2 AND #4	9
#6	#1 AND #3 AND #4	25
#7	#5 OR #6	0

(with Cochrane Library publication date from Jan 2021 to present)

PROSPERO – 2 SEP – 29 RESULTS

#1 (antenatal OR prenatal OR pregnan* OR matern* OR child health OR postnatal OR perinatal OR reproductive OR birth OR family plan* OR infant* OR mother* OR women OR woman OR ANC OR PNC):TI 7907

#2 (review OR systematic OR synthes*):TI 95012

#3 (utilis* OR utiliz* OR promot* OR uptake):TI 1441

#4 ((communit* OR public OR stakeholder OR local* OR social OR patient* OR consumer*) NEAR2 (mobiliz* OR mobilis* OR engage* OR participat* OR sensiti* OR communicat* OR messag* OR dialog* OR outreach OR promot* OR media OR capacity OR educat* OR interven* OR behavio*)):TI 697

#5 #2 AND #3 AND #1 147

#6 #1 AND #2 AND #4 13

#7 #5 OR #6 158
with date filter: 29

TOTAL = 178
IN COVIDENCE AFTER DUPLICATES = 124

Appendix 2: JBI Critical Appraisal

Author, Year	*Is review question clearly and explicitly stated?*	*Were inclusion criteria appropriate for review question?*	*Was search strategy appropriate?*	*Were sources and resources used to search for studies adequate?*	*Were criteria for appraising studies appropriate?*	*Was critical appraisal conducted by two or more reviewers independently?*	*Were there methods to minimize errors in data extraction?*	*Were methods used to combine studies appropriate?*	*Was likelihood of publication bias assessed?*	*Were recommendations for policy and/or practice supported by reported data?*	*Were specific directives for new research appropriate?*
George et al	Yes	Yes	Yes	Yes	Yes	Yes	Yes	Yes	Yes	Yes	Yes
Kearns et al	No	Unclear	Yes	Yes	No	No	No	Unclear	No	Yes	Yes
Kuhlmann et al	Yes	Yes	No	Yes	No	No	No	Yes	Yes	Yes	Yes
Lassi et al	Yes	Yes	Yes	Yes	Yes	Yes	Yes	Yes	Yes	Yes	Yes
Mangham-Jeffries et al	Unclear	Yes	Yes	Yes	Yes	Unclear	Yes	Yes	Yes	Yes	Yes
Marston et al	Yes	Yes	Yes	Yes	Unclear	Unclear	Unclear	Yes	Yes	Yes	Yes
Mbuagbaw et al	Yes	Yes	Yes	Yes	Yes	Yes	Yes	Yes	Yes	Unclear	Yes
Perry et al & Jennings et al	Yes	Yes	Yes	Yes	Unclear	Unclear	Yes	Yes	Yes	Yes	Yes
Prost et al	Yes	Yes	Yes	Yes	Yes	Unclear	Yes	Yes	Yes	Yes	Unclear
Sarkar et al	Yes	Yes	Yes	Yes	Yes	Unclear	Yes	Yes	Unclear	Yes	Unclear
Sharma et al	Yes	Yes	Yes	Yes	Yes	Yes	Yes	Yes	Yes	Yes	Yes
Wekesah et al	Yes	Yes	Yes	Yes	Unclear	Unclear	No	Yes	Yes	Yes	No
Beck et al	Yes	Yes	Unclear	Unclear	Unclear	Unclear	Unclear	Yes	Yes	Yes	Yes
Deshmukh et al	No	No	Unclear	Unclear	Yes	Yes	Unclear	Yes	Yes	Yes	No
George & Branchini	Yes	Yes	Unclear	Unclear	Yes	No	Yes	Yes	Yes	Yes	Yes

Gogia et al	Unclear	Yes	Unclear	Yes	Unclear	No	No	Yes	Yes	Yes	Yes
Muzyamba et al	Yes	Yes	Yes	Yes	Unclear	No	No	Yes	No	Yes	Yes
Parsekar et al	Unclear	Unclear	Unclear	Yes	Unclear	No	No	No	Unclear	Yes	No
Schiffman et al	Yes	Unclear	Unclear	Yes	No	No	Unclear	Yes	No	Yes	Yes
Singh et al	Yes	Unclear	Yes	Yes	Yes	No	Unclear	Yes	Yes	Yes	Yes
Takah et al	Yes	Yes	Yes	Yes	Yes	Unclear	Yes	Yes	Yes	Yes	Yes
Yuan et al	Yes	Yes	Yes	Yes	Yes	Yes	Yes	Yes	Yes	Yes	Yes

Appendix 3: Sustainable Development Goals (SDG) regions represented in reviews

STUDY	COUNTRIES	SDG REGIONS						
George et al	India, Uganda	Sub-Saharan Africa	Central and Southern Asia					
Kearns et al	Ethiopia, Nepal, Pakistan, Tanzania, Bangladesh, Kenya, USA, Australia, Netherlands, Malawi, Tanzania	Sub-Saharan Africa	Central and Southern Asia	Europe and North America				Oceania
Lassi et al	India, Pakistan, Bangladesh, Nepal, China, Vietnam, Syria, Malawi, Tanzania, Uganda, Ethiopia, Egypt, Kenya, South Africa, Zambia, Guatemala, Argentina	Sub-Saharan Africa	Central and Southern Asia		Latin America and the Caribbean	Eastern and South-Eastern Asia		
Mangham-Jeffries et al	LMICs (Bangladesh, Benin, Guinea, Burkina Faso, Cambodia, DRC, The Gambia, Honduras, India, Indonesia, Kenya, Malawi, Mozambique, Nepal, Niger, Nigeria, Papa New Guinea, Senegal, Uganda, Ukraine, Zambia)	Sub-Saharan Africa	Central and Southern Asia	Europe and North America	Latin America and the Caribbean	Eastern and South-Eastern Asia		
Marston et al	Bangladesh, Kenya, Malawi, Nepal, India	Sub-Saharan Africa	Central and Southern Asia					
Mbuagbaw et al	Argentina (2), Bangladesh (4), Brazil, Cuba (2), Eastern China, Ghana, Honduras, India (3), Laos, Malawi, Mexico (3), Mongolia, Nepal (2), Pakistan (3), Rwanda, Saudi Arabia, South Africa (2), Southern Tanzania, Thailand, Uganda, United Kingdom, USA (3), Vietnam, Zanzibar and Zimbabwe	Sub-Saharan Africa	Central and Southern Asia	Europe and North America	Latin America and the Caribbean	Eastern and South-Eastern Asia	Northern Africa and Western Asia	
Perry et al & Jennings et al	Global LMICs	Sub-Saharan Africa	Central and Southern Asia	Europe and North America	Latin America and the Caribbean	Eastern and South-Eastern Asia	Northern Africa and Western Asia	
Prost et al	Bangladesh, India, Malawi, Nepal	Sub-Saharan Africa	Central and Southern Asia					
Sarkar et al	India, Nepal, Malawi	Sub-Saharan Africa	Central and Southern Asia					
Kuhlmann et al	India, Burma, Pakistan, Tanzania, Argentina, DRC, Guatemala, Zambia, Cambodia, Bangladesh, Brazil, El Salvador, Peru, Honduras, Bolivia, Senegal, Ghana, Cameroon, Australia, Scotland, England	Sub-Saharan Africa	Central and Southern Asia	Europe and North America	Latin America and the Caribbean	Eastern and South-Eastern Asia		Oceania

Sharma et al	South Asia (Bangladesh, India, Nepal, Pakistan)		Central and Southern Asia					
Wekesah et al	Sub-Saharan Africa	Sub-Saharan Africa						
Beck et al	Sub-Saharran Africa (Kenya, Cameroon, Guinea Bissau, Madagascar, Liberia, Uganda, Nigeria, Malawi)	Sub-Saharan Africa						
Deshmukh et al	Tanzania, Cameroon, Ethiopia, Malawi, Zambia, Morocco, Uganda, Ghana, Sudan, India, Nepal, Bangladesh, Pakistan, Indonesia, Philippines, China, Afghanistan, Jordan, Palestine	Sub-Saharan Africa	Central and Southern Asia			Eastern and South-Eastern Asia	Northern Africa and Western Asia	
George & Branchini	India, Uganda, Angola, Ethiopia, Ghana, Kenya, Liberia, Mozambique, Tanzania, Bangladesh, Bolivia, DRC, Peru, Mexico, Cambodia, Canada, China, Honduras, Ghana, Nepal, Romania, Uruguay, China, Ghana, Pakistan, Kazakhstan, Argentina, Guatemala, South Africa	Sub-Saharan Africa	Central and Southern Asia	Europe and North America	Latin America and the Caribbean	Eastern and South-Eastern Asia		
Gogia et al	Pakistan, Bangladesh, India, Greece, Nepal, The Gambia	Sub-Saharan Africa	Central and Southern Asia	Europe and North America				
Muzyamba et al	Sub-Saharan Africa (Tanzania, Malawi, Kenya, Ethiopia, Angola, Sahel, Nigeria, Sudan, South Africa, Zambia)	Sub-Saharan Africa						
Parsekar et al	South Asia (Bangladesh, India, Nepal)		Central and Southern Asia					
Schiffman et al	India, Bangladesh, Pakistan, Nepal, Argentina, DRC, Guatemala, Zambia	Sub-Saharan Africa	Central and Southern Asia		Latin America and the Caribbean			
Singh et al	India		Central and Southern Asia					
Takah et al	Sub-Saharran Africa (Mozambique, Tanzania, Uganda, Malawi, Zambia, Kenya)	Sub-Saharan Africa						
Yuan et al	LMICs - Brazil, India, Guinea-Bissau, Bangladesh, Nepal, South Africa, Indonesia, Nigeria, Uganda, Philipines, Tanzania, Zambia, China, Colombia	Sub-Saharan Africa	Central and Southern Asia		Latin America and the Caribbean	Eastern and South-Eastern Asia		
	**COUNT:**	**19**	**18**	**7**	**8**	**8**	**3**	**2**

Appendix 4: Key findings as reported by reviews

Author	Geographic Setting	# of Studies in Review	Outcome of Interest	# of Studies with CM + ANC/ PNC	Stated Objective/ Question	CM Activities [as reported in review]	Key Findings/Major Themes
George et al 2015	LMICs (India, Uganda)	4	ANC & early ANC	4 (1 early ANC)	What interventions to promote awareness of human rights/sexual and reproductive rights /right to access to quality care are effective in increasing demand for and use of health care to improve maternal and newborn health outcomes?	public meetings, events with community and staff, household visits, women's groups, awareness campaigns, local leader meetings	Four studies demonstrated increase in ANC use, though one was not statistically significant.<br><br>Antenatal examinations increased 30%, $p<0.001$ (Pandey), registration for ANC within 3 months of pregnancy increased from 31.4% to 54.3% and 17% to 41.8% (Ganju) and 95.8% received 1 ANC visit (vs 90.3%), $p<0.001$ and 72.5% received 3+ ANC visits (vs 61.0%), $p<0.001$ (Sinha).<br><br>Average number of ANC visits provided at a facility per month were 5.5, but this was not a statistically significant difference (Bjorkman).
Kearns et al 2016	Global (Australia, Bangladesh, Ethiopia, Kenya, Malawi, Nepal, Netherlands, Pakistan, Tanzania, USA)	8	ANC & PNC	3	This study focused on identifying and analysing innovative approaches aimed at improving ANC and PNC and drawing potentially generalisable lessons from them.	women's groups, lady healthworkers, committees with local leaders	Women's groups can improve accessibility and acceptability of ANC and PNC by building consensus, creating a support network, and providing relevant health and pregnancy related information.
Kuhlmann et al 2016	Global (Argentina, Australia, Bangladesh, Bolivia, Brazil, Burma, Cambodia, Cameroon, DRC, El Salvador, England, Ghana,	32	ANC	16 (1 early ANC)	To identify and assess the mechanisms of effect and the quality of evidence for the effectiveness of a spectrum of interventions along the three stages of CM across five sets of SRMH outcomes: maternal health and obstetric care,	home visits for education; peer-facilitated and community/staff led participatory learning groups	One study in Pakistan reported more pregnant women received ANC post-intervention (from 65.9% to 79.2%) while another reported that 90.8% of pregnant women received ANC by a trained TBA in the intervention communities.<br><br>An additional study in Burma reported significant increases in at least one ANC visit (from 39.3% to 71.8%, PRR=1.83, 95% CI: 1.64-2.04) as well as in 4+ ANC visits (from 6.7% to 34.4%, PRR= 2.06, 95% CI: 1.72-2.47).

Author	Geographic Setting	# of Studies in Review	Outcome of Interest	# of Studies with CM + ANC/ PNC	Stated Objective/ Question	CM Activities [as reported in review]	Key Findings/Major Themes
	Guatemala, Honduras, India, Pakistan, Peru, Scotland, Senegal, Tanzania, Zambia)				antenatal care (ANC) and birth planning, postnatal/newborn care, breastfeeding, and birth spacing/family planning.		A study in India reported significantly greater increases in 3 ANC visits in the intervention community compared to the control (3.6% to 12.2% versus 6.9% to 9.0%, p<0.001).<br><br>A multi-country study (Argentina, DRC, Guatemala, India, Pakistan, and Zambia) reported a significant increase in primigravida women who booked ANC visits early in pregnancy (18.75% to 56.9%, p<0.001) but a nonsignificant increase in those who had at least 4+ ANC visits during pregnancy.<br><br>A study in Cambodia reported an increase of 22% in ANC utilization (1015 women to 1241 women).<br><br>A study in Honduras reported that ANC visits increased from 180/month to 258/month and that women attending 4+ ANC visits increased (from 62% to 87%). A study in Nepal reported that women in the intervention communities were more likely to have had any ANC visit (aOR = 2.82, 95% CI: 1.41, 5.62).<br><br>A study in Bangladesh reported a significant increase in at least 1 ANC visit (48% to 72%).<br><br>Studies in Bangladesh, Bolivia and India reported increases in ANC utilization but it was not statistically significant<br>- India: any ANC visits (aOR = 1.60, 95% CI: 0.65, 3.92) or in 3 ANC visits (aOR =0.69, 95% CI: 0.37, 1.26);<br>- Bangladesh: any ANC (aRR = 0.91, 95% CI: 0.76, 1.09) or 4 ANC visits (aRR = 0.79, 95% CI: 0.46, 1.37)
Lassi et al 2019	LMICs (Argentina, Bangladesh, China, Egypt, Ethiopia,	33	ANC	18	To compare a community health educational strategy versus no strategy or the existing approach	educational interventions - one-to-one & group counseling	Random-effects model analysis studies found community health education interventions increased ANC utilization by 16% (RR 1.16, 95% CI 1.11 to 1.22;18 studies; n = 307,528; I2 = 96%; Chi2 P < 0.00001).

Author	Geographic Setting	# of Studies in Review	Outcome of Interest	# of Studies with CM + ANC/ PNC	Stated Objective/ Question	CM Activities [as reported in review]	Key Findings/Major Themes
	Guatemala, India, Kenya, Malawi, Nepal, Pakistan, South Africa, Syria, Tanzania, Uganda, Vietnam, Zambia)				to health education on maternal and newborn care in LMICs, as imparted to mothers or their family members specifically in community settings during the antenatal and/or postnatal period, in terms of effectiveness for improving neonatal health and survival (i.e. neonatal mortality, neonatal morbidity, access to health care, and cost)		Additionally, receiving both one-to-one and group counselling saw an increase of ANC utilization by 21% (RR 1.21, 95% CI 1.07 to 1.37; 5 studies; n = 51,352; I2 = 97%; Chi2 P < 0.00001)
Mangham-Jeffries et al 2014	LMICs (Bangladesh, Benin, Guinea, Burkina Faso, Cambodia, DRC, The Gambia, Honduras, India, Indonesia, Kenya, Malawi, Mozambique, Nepal, Niger, Nigeria, Papa New Guinea, Senegal, Uganda, Ukraine, Zambia)	48	ANC	2	To identify studies that report on the cost-effectiveness of strateg ies to improve the utilization and/or provision of maternal and newborn health care in low-income and lower-middle-income countries.	women's groups, home visits	One study in Cambodia reported a 22% increase in ANC.<br><br>An additional study in Bangladesh reported numbers of new ANC users.

Author	Geographic Setting	# of Studies in Review	Outcome of Interest	# of Studies with CM + ANC/ PNC	Stated Objective/ Question	CM Activities [as reported in review]	Key Findings/Major Themes
Marston et al 2013	LMICs (Bangladesh, Kenya, Malawi, Nepal, India)	15	ANC	7	Examining the effectiveness of community participation interventions in maternal and newborn health, asking: did participation improve outcomes?	Women's groups (Nepal, Bangladesh, India, Malawi); evidence-based dialogue model (Kenya); participatory methodologies (Nepal)	A women's group intervention in Nepal was reported to have a positive effect on ANC uptake (AOR=2.82, 95% confidence interval 1.41–5.62).<br><br>An additional study on youth in Nepal reported overall use of antenatal facilities actually decreased even though the participatory approach empowered the target population and increased the community's demand for information.<br><br>Two studies in India as well as a study on participatory dialogue in Kenya did not report a statistically significant increase in ANC.<br><br>A study in Malawi reported marginally higher odds of ANC in a health facility (AOR=1.44, 95%CI 1.00-2.13).
Mbuagbaw et al 2015	Global (Argentina, Bangladesh, Brazil, Cuba, Eastern China, Ghana, Honduras, India, Laos, Malawi, Mexico, Mongolia, Nepal, Pakistan, Rwanda, Saudi Arabia, South Africa, Southern Tanzania, Thailand, Uganda, United Kingdom,	34	16 (1 early ANC)	23	To assess the effects of health system and community interventions for improving coverage of antenatal care and other perinatal health outcomes.	Mass media campaigns. Social mobilisation. Information-education-communication (IEC).	This synthesis considered outcomes when populations received one or multiple community interventions.<br><br>In populations that received one intervention versus none, ANC coverage of 4+ visits showed "marginal results favoring the interventions and substantial heterogeneity (average odds ratio (OR) 1.11, 95% confidence interval (CI) 1.01 to 1.22; studies = 10; 45,022 women" when using an intra-cluster correlation coefficient of 0.02. Pooled results for ANC coverage of 1+ visits demonstrated a marginal improvement but also high levels of heterogeneity (average OR 1.68, 95% CI 1.02 to 2.79; studies = 6).<br><br>In populations that received a combination of interventions versus none, there was no clear difference in ANC coverage of 4+ visits, however for ANC coverage of 1+ visit, women in the intervention arms had higher odds of attending at least one ANC session (average OR 1.79, 95% CI 1.47 to 2.17; studies = 5). One study reported that a combined intervention did not encourage more women to attend ANC earlier

Author	Geographic Setting	# of Studies in Review	Outcome of Interest	# of Studies with CM + ANC/ PNC	Stated Objective/ Question	CM Activities [as reported in review]	Key Findings/Major Themes
	USA, Vietnam, Zanzibar, Zimbabwe)						in pregnancy (OR 0.83, 95% CI 0.47 to 1.47; studies = 1).
Perry et al 2017 & Jennings et al 2017	LMICs	700	ANC	maternal health - 152;<br>ANC - 37	Community-based primary health care to improve MNCH in LMICs: (1) What kinds of projects were implemented? (2) What were the outcomes of these projects? (3) What kinds of implementation strategies were used? (4) What are the implications of these findings?	four common implementation interventions: home visits, community case management, participatory women's groups, mobile teams at health facilities (about health system/access, not CM)	34 studies reported increases in ANC, three studies reported no change (or no statistically significant change) in ANC, no studies reported a decrease in ANC [chapter 2]
Prost et al 2013	LMICs (Bangladesh, India, Malawi, Nepal)	7	ANC - 6;<br>PNC - 5	6	To assess the effect of women’s groups practising participatory learning and action, compared with usual care, on maternal mortality, neonatal mortality, and stillbirths in low-resource settings	women's groups	Two studies reported on how women's groups significantly increased ANC uptake.<br><br>Four additional studies did not find significant differences in ANC uptake.<br><br>One study reported an increase in postpartum checks.<br><br>Two studies reported a statistically insignificant increase in care-seeking in case of a postpartum problem for the mother, while four reported a statistically insignificant increase in care-seeking in case of a postpartum problem for the newborn.

Author	Geographic Setting	# of Studies in Review	Outcome of Interest	# of Studies with CM + ANC/ PNC	Stated Objective/ Question	CM Activities [as reported in review]	Key Findings/Major Themes
							One study reported a statistically significant increase in care-seeking practices in case of a newborn's health problem.
Sarkar et al 2015	LMICs (India, Malawi, Nepal)	8	ANC & PNC	5	To identify and analyze effective community based interventions for improving the reproductive health outcomes of young married women (aged 15–24 years) that can be delivered in a resource-constrained setting.	individual and group conseling, community education campaigns, group training, advocacy workshops, sensitization, street plays and drama, youth groups, events	Three of the initiatives demonstrated improvements in ANC - ANC check-ups by first time mothers increased in FTP (in Diamond Harbor, Baseline: 33%, End line: 53%), ACQUIRE (Baseline: 29%, End line: 50%) and REWARD (Mothers Groups: Baseline: 20%, End line: 54%).<br><br>These three initiatives also reported on improving PNC, but only two of them (FTP and ACQUIRE) showed a significant improvement in first-time mothers receiving PNC check-ups within 6 weeks of postpartum (effect ranged 10-39%).
Sharma et al 2018	South Asia (Bangladesh, India, Nepal, Pakistan)	11	ANC	8	To compare the effectiveness of interventions to promote family and community participation in maternal health care against standard health care and health service led programs on the outcomes: indicators of maternal health care knowledge improvement, maternal health care utilisation (antenatal care, facility birth, skilled birth attendant use), and maternal mortality in rural-remote regions of the South Asian countries,	community mobilisers deliver MH education: home care and community care; HCWs community education; women's MH education group; women and men's MH health care education groups	Community interventions increased attendance of 1+ ANC visit by an average of 19% (RR 1.19, 95% CI 1.06 to 1.33; participants = 75,737; studies = 8; I2 = 58%) compared with control.

Author	Geographic Setting	# of Studies in Review	Outcome of Interest	# of Studies with CM + ANC/ PNC	Stated Objective/ Question	CM Activities [as reported in review]	Key Findings/Major Themes
					Bangladesh, India, Nepal, and Pakistan.		
Wekesah et al 2016	Sub-Saharan Africa	73	ANC - 6; PNC - 1	8	A systematic review of published evidence on non-drug interventions that reported effectiveness in improving outcomes and quality of care in maternal health in sub-Saharan Africa.	1. Community mobilization through women's groups; 2. Skilled birth attendants; 3. Training health extension workers; 4. Training and deployment of community health development agents; 5. Traditional birth attendants (TBAs); 6. Family and community members meetings on health care; 7. Trained volunteers to provide health care at the community; 8. Village health teams; 9. Peer mentors who women are living with HIV	One study in Kenya reported a significant increase in ANC from 39% to 62%.<br><br>A study in Tanzania found women in the intervention arm were 22.7% (versus 2.2%) more likely to attend PNC within the first 48 hours.

Author	Geographic Setting	# of Studies in Review	Outcome of Interest	# of Studies with CM + ANC/ PNC	Stated Objective/ Question	CM Activities [as reported in review]	Key Findings/Major Themes
Beck et al 2019	Sub-Saharan Africa (Cameroon, Guinea Bissau, Kenya, Liberia, Madagascar, Malawi, Nigeria, Uganda)	19	ANC	2	To gain an understanding of how CM interventions for maternal and child health in sub-Saharan Africa impact the empowerment of individuals	None listed	Two studies demonstrated CM increased care-seeking behaviours, two studies demonstrated increased number of ANC visits.
Deshmukh et al 2020	LMICs (Afghanistan, Bangladesh, Cameroon, China, Ethiopia, Ghana, India, Indonesia, Jordan, Malawi, Morocco, Nepal, Pakistan, Palestine, Philippines, Sudan, Tanzania, Uganda, Zambia)	47	PNC	*unknown*	To profile the determinants of PNC service provision and utilization patterns in a comprehensive manner as available in the published literature in low resource settings.	social mobilization as an opportunity for education/awareness	The reviewers identified social mobilization as an opportunity to increase awareness of PNC services and health complications in the nexus framework they applied to the included studies.
George & Branchini 2017	LMICs (Angola, Argentina, Bangladesh, Bolivia, Cambodia, Canada, China, DRC, Ethiopia, Ghana, Guatemala, Honduras,	26	ANC	4	Examine the diverse range of initiatives that promote awareness of rights to quality maternity care services to derive the common principles and processes related to stakeholder experiences and implementation factors	public meetings, committees, information dissemination	Three studies demonstrated an increase in ANC uptake with CM initiatives present (two in India, one in Uganda).<br><br>An additional study in India demonstrated an increase in ANC uptake but it was not statistically significant.

Author	Geographic Setting	# of Studies in Review	Outcome of Interest	# of Studies with CM + ANC/ PNC	Stated Objective/ Question	CM Activities [as reported in review]	Key Findings/Major Themes
	India, Kazakhstan, Kenya, Mexico, Mozambique, Nepal, Nigeria, Pakistan, Peru, Romania, South Africa, Tanzania, Uganda, Uruguay)				to guide future efforts in this area.		
Gogia et al 2011	LMICs (Bangladesh, The Gambia, Greece, India, Nepal, Pakistan)	13	ANC	5	To assess the effect of community based neonatal care by community health workers (CHWs) on neonatal mortality rates in resource-limited settings.	group meetings, community health committees, education sessions, participatory action learning cycles	One study demonstrated improvement in at least 1 ANC visit.
Muzyamba et al 2017	Sub-Saharan Africa (Angola, Ethiopia, Kenya, Malawi, Nigeria, Sahel, South Africa, Sudan, Tanzania, Zambia)	14	ANC	3	To provide an overview of the empirical evidence on the role of community mobilization in maternal care provision to women living with HIV in sub-Saharan Africa	Group/peer support for pregnant women/new mothers	Three studies reported that peer support for women with HIV increased access to health resources, including ANC.
Parsekar et al 2020	South Asia (Bangladesh, India, Nepal)	*Not stated*	ANC	1	To collate the evidence on the current status of “Reproductive, Maternal, Newborn, Child and Adolescent Health” (RMNCAH) and related Behaviour	information, education, communication/ behaviour change communication initiative	*This narrative review described the strategies used by one IEC initiative in Nepal but did not report on the outcomes.*

Author	Geographic Setting	# of Studies in Review	Outcome of Interest	# of Studies with CM + ANC/ PNC	Stated Objective/ Question	CM Activities [as reported in review]	Key Findings/Major Themes
					Change Communication (BCC) interventions in RMNCAH in South Asia		
Schiffman et al 2010	LMICs (Argentina, Bangladesh, DRC, Guatemala, India, Nepal, Pakistan, Zambia)	9	ANC	4	(1) to systematically identify largescale, controlled studies that test a CBIP with a primary focus on Family-Community Care interventions because they have a greater potential impact than Outreach and Clinical Care interventions, (2) to describe the positive and negative findings of the studies identified, and (3) to summarize the lessons learned.	community health committees, group meetings, participatory learning activities, folksongs	*This review focused on presenting mortality-related outcomes and did not report on the outcomes related to changes in care seeking.*
Singh et al 2021	India	66	ANC	1	To synthesize the impacts of public health programs on uptake of maternal and child health services as well as the short-term/ intermediate and long-term outcomes.	ASHA health awareness	*This review presented one study with ASHAs which demonstrated an increase in ANC.*
Takah et al 2019	Sub-Saharan Africa (Kenya, Malawi, Mozambique, Tanzania, Uganda, Zambia,)	8	ANC	1	1. To determine the interventions/approaches used to improve male partner involvement within the context of PMTCT, specifically hospital delivery services by	community meetings (talks, music, dance, dramas)	*This review did not present outcomes related to ANC/PNC care seeking.*

Author	Geographic Setting	# of Studies in Review	Outcome of Interest	# of Studies with CM + ANC/ PNC	Stated Objective/ Question	CM Activities [as reported in review]	Key Findings/Major Themes
					pregnant women living with HIV in sub-Saharan Africa.<br>2. To determine the impact of these approaches on the utilization of hospital delivery services by pregnant women living with HIV in sub-Saharan Africa.		
Yuan et al 2014	LMICs (Bangladesh, Brazil, China, Colombia, Guinea-Bissau, India, Indonesia, Nepal, Nigeria, Philippines, South Africa, Tanzania, Uganda, Zambia)	22	ANC	2	To collect evidence about the differential effects of interventions on different sociodemographic groups in order to identify interventions that were effective in reducing maternal or child health inequalities.	participatory women's group	One intervention in Bangladesh demonstrated a reduction in income inequalities in access to ANC.

Appendix 5: Additional evidence reported in reviews to support Downe et al. qualitative evidence synthesis

DOWNE ET AL. FINDINGS	ADDITIONAL INSIGHT	REFERENCE
	SOCIO-CULTURAL CONTEXT	
W1. INFLUENCE OF TRADITIONAL BELIEFS	Sixteen studies described how community contexts, cultural beliefs, traditions, and norms influence women's abilities to seek care during postnatal period.	Deshmukh et al
	One included study used stakeholder problem-solving meetings to determine action/priorities.	Mbuagbaw et al
	Review operationalized community mobilization in the included studies along three principles: "[1] collaboration between community and professionals in designing maternal care initiatives, [2] use of indigenous resources in maternal care provision (e.g. TBA), [3] reliance on peer support for pregnant women and new mothers."	Muzyamba et al
W2. INFLUENCE OF OTHERS	Studies described how community biases and expectations could be a barrier that was overcome through separate stakeholder meetings, discussions around male participation or resistance, identification of local champions. These community conflicts could also be influenced by inherent divisions in the community for example, along political party lines. The influence of others, such as family or friends, was an important component of implementation. Peers through women support groups for example provided "space for social bonding, changing social norms and building self-esteem."	George & Branchini
P1. CO-OPERATION WITH INFLUENTIAL COMMUNITY MEMBERS	Studies described community mobilization "is reliant on components related to the incorporation of existing structures, partnership building, and local context." These strategies used existing community groups and leaders, health workers, and traditional birth attendants. It was also imperative to build partnerships at local, regional, and/or national levels (with government and nongovernment officials as well as local leaders and chiefs) in order to promote sustainable community mobilisation initiatives.	Beck et al
	The review described an initiative where information that was share with women's groups was then also taken to the health system level through local elected representatives in order to collaborate on local action plans to resolve issues.	George et al
	Studies described how community biases and expectations could be a barrier that was overcome through separate stakeholder meetings, discussions around male participation or resistance, identification of local champions. These community conflicts could also be influenced by inherent divisions in the community for example, along political party lines. Studies emphasized gaining buy-in and the involvement of community leaders prior to initiating any community initiatives, including government and health systems representatives. The successful projects involved community organizers who were trusted by the local community and included strategic alliances across civil society and government representatives. Two studies highlighted the importance of gender balance in organizers and peer educators in order to reach both men and women in the community.	George & Branchini
	Local leaders were used to promote ANC attendance through community mobilization strategies.	Mangham-Jeffries et al
	Review operationalized community mobilization in the included studies along three principles: "[1] collaboration between community and professionals in designing maternal care initiatives, [2] use of indigenous resources in maternal care provision (e.g. TBA), [3] reliance on peer support for pregnant women and new mothers."	Muzyamba et al
	Potential strategies included engaging community leaders to mobilize the community and the formation of village of health committees.	Perry et al & Jennings et al
	One study described involving local male champions to influence acceptability and higher uptake of hospital delivery.	Takah et al
	Involvement of "technical leaders and policy makers" to develop standards that would be nationally acceptable.	Wekesah et al
P2. TRADITIONAL, SOCIETAL AND	Community mobilization strategies were influenced by local context such as "socio-cultural preferences or beliefs, political influence, and/or environmental, geographical, or historical factors."	Beck et al

COMMUNITY NORMS, PRACTICES AND BELIEFS	Studies described how community biases and expectations could be a barrier that was overcome through separate stakeholder meetings, discussions around male participation or resistance, identification of local champions. These community conflicts could also be influenced by inherent divisions in the community for example, along political party lines. Studies emphasized gaining buy-in and the involvement of community leaders prior to initiating any community initiatives, including government and health systems representatives. The successful projects involved community organizers who were trusted by the local community and included strategic alliances across civil society and government representatives. Two studies highlighted the importance of gender balance in organizers and peer educators in order to reach both men and women in the community.	George & Branchini
	One study highlighted the importance of considering cultural norms in delivering financial incentives to women.	Kearns et al
W6. GENDER ISSUES - FINANCIAL DEPENDENCE ON HUSBAND	The involvement of men/male partners in ANC was stressed by Tanzania's Ministry of Health and Social Welfare in order to improve women's access to care and therefore improve maternal, newborn, and child health. One study highlighted the importance of considering cultural norms in delivering financial incentives to women.	Kearns et al
	Community engagement activities can empower women through education and awareness, women's groups, and financial programs such as women's microcredit and savings groups.	Perry et al & Jennings et al
W8. GENDER ISSUES - GENDER OF HEALTH CARE PROVIDER	Two studies highlighted the importance of gender balance in organizers and peer educators in order to reach both men and women in the community.	George & Branchini
	Studies used Lady Health Workers to provide basic care to women as well as health education in the community.	Kearns et al
W9. GENDER ISSUES - WOMEN'S FREEDOM OF MOVEMENT	Gender norms could affect women's education as well as mobility, which influenced their participation in interventions and care-seeking behaviour.	Sarkar et al
	SERVICE PHILOSOPHY, DESIGN, AND PROVISION	
W10. POOR INFRASTRUCTURE	Studies reported how programming, human resource skills, supply chain management, and lack of transportation contributed to poor quality ANC and PNC.	Kearns et al
W11 & P3. PROXIMITY OF CLINIC	26 studies described accessibility as household-level barriers.	Deshmukh et al
	Studies described how distance from a facility could prevent timely care in remote areas and that making services and referral hospitals accessible improved health and wellbeing.	Kearns et al
P4. AVAILABILITY OF TRANSPORT	One study reported how making transportation available increased the accessibility of care.	George et al
	Studies reported how programming, human resource skills, supply chain management, and lack of transportation contributed to poor quality ANC and PNC.	Kearns et al
	Collaborative design was used to create initiatives to improve access to care - including transportation during obstetric emergencies.	Muzyamba et al
W12. INDIRECT COST OF SERVICES	One study provided free delivery care as well as coverage for transportation costs.	Parsekar et al
	14 studies described financial issues as household-level barriers.	Deshmukh et al
	Three studies examined reducing the costs of maternal care through removing user fees, introducing fixed charges, or using vouchers to cover care and transport costs.	Mangham-Jeffries et al
	This review described the “Reproductive Health Vouchers Programme” or DSF programme launched by the MOHFW with WHO support. This program provided ANC, PNC, and emergency care as well as cash incentives.	Parsekar et al
	This review listed microcredit programs for women as a way to combat financial barriers.	Perry et al & Jennings et al
	12 studies reported demand-side financial incentives such a user fee exemptions, cost-sharing programs, and vouchers for care, facility-based deliveries and other indirect costs such as transportation.	Wekesah et al
	Payment schemes and initiatives led to increased numbers of institutional deliveries as well as improvements in ANC services.	Wekesah et al
W17. FLEXIBILITY OF APPOINTMENTS	One study in Andhra Pradesh reported that auxiliary nurse midwives were more available for appointments and responsive.	George et al

P12. SHORTAGE OF STAFF	The review reported multiple studies where healthcare workers cited difficult work environments, staff shortages, limited supplies and resources, and large patient loads.	George & Branchini
	Health systems challenges such as staff shortages and limited supplies and resources were barriers to appropriate ANC delivery. Studies reported how programming, human resource skills, supply chain management, and lack of transportation contributed to poor quality ANC and PNC.	Kearns et al
P13. AVAILABILITY OF RESOURCES	Three studies described how providers believed available resources and incentives enabled provision of PNC.	Deshmukh et al
	The review reported multiple studies where healthcare workers cited difficult work environments, staff shortages, limited supplies and resources, and large patient loads.	George & Branchini
	Health systems challenges such as staff shortages and limited supplies and resources were barriers to appropriate ANC delivery.	Kearns et al
	One study described strengthening the capacity of the health system to manage referrals through improved equipment and training.	Schiffman et al
P14. STAFF WORKING CONDITIONS	The review reported multiple studies where healthcare workers cited difficult work environments, staff shortages, limited supplies and resources, and large patient loads.	George & Branchini
P15. STAFF TRAINING	Healthcare provider's knowledge and skills as well as availability of supplies in the health system adversely affected PNC utilization.	Deshmukh et al
	Studies reported using ongoing refresher trainings for healthcare providers as well as promoting team approaches to care and strengthening interpersonal skills.	George & Branchini
	One of the case studies presented emphasized programmes that strengthened increased provider training and referral networks and resulted in increases in PNC and postpartum contraception uptake.	Kearns et al
	Studies reported healthcare worker trainings improved knowledge, confidence, and overall availability and quality of emergency obstetric care. Specifically, in Burkina Faso, increased trainings, referral systems, and available resources and supplies increased facility-based deliveries as well as first ANC visits.	Wekesah et al
P16. NEED FOR MANAGEMENT SUPPORT	Studies reported how programming, human resource skills, supply chain management, and lack of transportation contributed to poor quality ANC and PNC.	Kearns et al
WHAT MATTERS TO WOMEN & STAFF (PERSONALISED SUPPORTIVE CARE)		
W19. SOCIAL & COMMUNITY SUPPORT - INVOLVEMENT OF THE COMMUNITY	A study described how messages were tailored based on cultural norms in the community as well as relatable metaphors.	Beck et al
	The review described an initiative where information that was share with women's groups was then also taken to the health system level through local elected representatives in order to collaborate on local action plans to resolve issues.	George et al
	Multiple studies emphasized the importance of "understanding women's realities and what matters most to communities." This includes involving community members in the design of interventions as well as understanding the local context and consideration of stakeholders.	George & Branchini
	Women's groups used participatory learning and action cycles and trained birth attendants and community health workers were drawn from the local community.	Gogia et al
	In one study, Lady Health Workers not only provided care but also served as a liaison between the community and the health facilities.	Kearns et al
	Community mobilization interventions developed by health or research staff build off of existing groups in the communities "in order to capitalize on the strength of existing support systems and social networks." Peer-facilitated learning and community dialogues organized in the community empowered community members to participate in programming and make decisions.	Kuhlmann et al
	Educational interventions at the community level increased ANC uptake.	Lassi et al
	Local community members were involved to identify priorities and implement solutions, such as supplying clean delivery kits and establishing community funds.	Mangham-Jeffries et al

	Multiple studies referred to the Warmi project women's groups which used participatory action research to have local communities/women identify and prioritise local problems and solutions.	Marston et al
	Communities were involved in problem identification and subsequent solution implementation. Examples of programs included creating community funds for care and providing transportation in case of obstetric emergencies.	Marston et al
	One study used regular meetings with community stakeholders to identify problems and potential solutions.	Mbuagbaw et al
	Review operationalized community mobilization in the included studies along three principles: "[1] collaboration between community and professionals in designing maternal care initiatives, [2] use of indigenous resources in maternal care provision (e.g. TBA), [3] reliance on peer support for pregnant women and new mothers." This collaboration in designing initiatives improved assess to treatment through initiatives such as providing transportation for pregnant women in case of obstetric emergencies.	Muzyamba et al
	Projects could be implemented by not only community health workers but also government professionals and trained community members. Programs with the community included the formation of community or village health committees and other community groups, along with the involvement of local leaders. Communities could also be engaged in not only the planning and implementation of the program, but also the evaluation through sharing data and participatory rural appraisals. The review also reported that "81% of the projects engaged communities in project implementation, and more than half promoted partnerships between the community and the health program, promoted the use of local resources, or promoted community empowerment."	Perry et al & Jennings et al
	Community groups in various programs implemented social mobilization activities which included special events and celebrations for the whole community as a form of health education/awareness.	Sarkar et al
	In one study, community health committees made up of local volunteers supported Lady Health Workers by holding regular group education sessions.	Schiffman et al
	One study had village volunteers or, "community resource people," identify pregnant women to encourage them to attend community meetings and seek ANC and PNC.	Schiffman et al
	One study that involved the community through a singing intervention to disseminate health care messaging about maternal health.	Sharma et al
	Communicating with the community and implementing community mobilization activities (such as dramas and music as well as visiting male partners at bars, football games, and churches) encouraged trust between the community and health care workers.	Takah et al
W20. SOCIAL & COMMUNITY SUPPORT - PEER SUPPORT	Family and friends were an important component of sustainable implementation of peer-based learning, for example. Peers through support groups or women provided "space for social bonding, changing social norms and building self-esteem."	George & Branchini
	One study described how local women held trainings on maternal health rights within the community to build "women's leadership capacity."	George & Branchini
	Women's groups used participatory learning and action cycles and trained birth attendants and community health workers were drawn from the local community.	Gogia et al
	Group care models for pregnant or postpartum women (or the entire community) fostered empowerment and provided support. These groups could provide ANC but largely were an avenue for women to come together, discuss local issues and solutions, and provide a support network during pregnancy. These groups have improved accessibility and acceptability of ANC and PNC and resulted in improved maternal and neonatal health outcomes as well as provider and patient satisfaction.	Kearns et al
	Five studies in Bangladesh, India, Malawi, and Nepal reported that women's groups improved MNH. Each program involved training local women to facilitate the monthly women's group meetings.	Mangham-Jeffries et al
	Multiple studies referred to the Warmi project women's groups which used participatory action research to have local communities/women identify and prioritise local problems and solutions.	Marston et al
	A women's group in Nepal based on the Warmi methodology had a positive effect on ANC uptake.	Marston et al
	Multiple studies used participatory women's groups.	Mbuagbaw et al
	The review operationalized community mobilization in the included studies along three principles: "[1] collaboration between community and professionals in designing maternal care initiatives, [2] use of indigenous resources in maternal care provision (e.g. TBA), [3] reliance on peer support for pregnant women and new mothers." Women's groups were used as a form of peer support to provide emotional, physical, social, and psychological support. Multiple studies found this peer support contributed to increases in health-resource utilisation.	Muzyamba et al

	This review reported that participatory women's groups were a common strategy to engage women and their families. These groups were led by trained facilitators and were used to provide "empowerment and education." There are multiple approaches to facilitating these women's groups including: participatory action-learning cycle, Care Groups, and education sessions.	Perry et al & Jennings et al
	This review hypothesized that women's groups improve birth outcomes by building "capacities of communities to organise and mobilise to take individual, group, and community action to address the structural and intermediary determinants of health." Women's group facilitators were local women who were trained to facilitate the group meetings, rather than health care workers. These groups were associated with increased ANC uptake.	Prost et al
	Peer support strategies have become more popular to improve maternal health care uptake. One study reported volunteer peer counsellor health education in women's groups decreased maternal mortality.	Wekesah et al
	Women's participatory groups increased awareness and demand for maternal health services and provided an opportunity for women to discuss and identify health challenges.	Yuan et al
P18. SOCIAL & COMMUNITY SUPPORT - SOCIAL SUPPORT FOR WOMEN	Women's groups used participatory learning and action cycles and trained birth attendants and community health workers were drawn from the local community.	Gogia et al
	This review hypothesized that women's groups improve birth outcomes by building "capacities of communities to organise and mobilise to take individual, group, and community action to address the structural and intermediary determinants of health."	Prost et al
W22. INDIVIDUALISED CARE - WOMEN-CENTRED CARE	Two case studies described increased uptake and satisfaction in care by supporting care-seeking behaviours. One of these programs also credited high patient satisfaction to the emphasis on patient-centred care.	Kearns et al
	One study reported increases in first ANC visits as well as the "quality of women-friendly services and satisfaction of women" as a result of community-based care.	Wekesah et al
W26. AUTHENTIC AND KIND STAFF	Six studies described that positive interactions between health workers and mothers increased ANC attendance and trust.	Deshmukh et al
P20. STAFF ATTITUDE	This review described how providers supported attitudinal changes. For example, while some challenges are " beyond their control," there was a recognized "need for a change in mindsets on their behalf."	George & Branchini
WHAT MATTERS TO WOMEN & STAFF (INFORMATION & SAFETY)		
W27. ANC AS A SOURCE OF KNOWLEDGE AND INFORMATION	In evaluating knowledge, awareness, and practices, it is clear that cultural beliefs and myths influence individual beliefs as well.	Beck et al
	In Tanzania, education and counselling activities at ANC visits included physiological and emotional components during pregnancy as well as postpartum care. Additionally, providers are trained on how to deliver this information rather than run through a checklist of facts.	Kearns et al
	In the quantitative synthesis, delivering education during ANC or during both ANC and PNC periods reduce early neonatal mortality.	Lassi et al
	One study described ANC coverage that included iron/folic acid supplements along with anaemia related health education.	Perry et al & Jennings et al
W31. ANC AS A SOURCE OF MEDICAL SAFETY	This review presented studies describing other maternal health objectives such as iron and iron-folic acid conception as well as tetanus toxoid vaccination coverage.	Kuhlmann et al
	Multiple studies described antenatal care as an avenue for additional, such as through the distribution of mosquito nets or HIV/syphilis testing. Additionally, improving MNH care through community health workers and home-based care volunteers was associated with additional malaria prophylaxis as well as vitamin and supplement distribution.	Mangham-Jeffries et al
	One study described ANC coverage that included iron/folic acid supplements along with anaemia related health education.	Perry et al & Jennings et al
